# Testing the Antimicrobial Characteristics of Wood Materials: A Review of Methods

**DOI:** 10.3390/antibiotics9050225

**Published:** 2020-05-01

**Authors:** Muhammad Tanveer Munir, Hélène Pailhories, Matthieu Eveillard, Mark Irle, Florence Aviat, Laurence Dubreil, Michel Federighi, Christophe Belloncle

**Affiliations:** 1 Laboratoire Innovation Matériau Bois Habitat Apprentissage (LIMBHA), Ecole Supérieure du Bois, 7 rue Christian Pauc, 44306 Nantes, France; tanveer.munir@esb-campus.fr (M.T.M.); mark.irle@esb-campus.fr (M.I.); 2 Laboratoire HIFIH, UPRES EA3859, SFR 4208, Université d’Angers, 49933 Angers, France; helene.pailhories@chu-angers.fr; 3 Laboratoire de bactériologie, CHU Angers, 49933 Angers, France; maeveillard@chu-angers.fr; 4 CRCINA, Inserm, Université de Nantes and Université d’Angers, 44200 Nantes, France; 5 Your ResearcH-Bio-Scientific, 307 la Gauterie, 44430 Le Landreau, France; florenceaviat@gmail.com; 6 PAnTher, Oniris, INRA, Université Bretagne Loire, F-44307 Nantes, France; laurence.dubreil@oniris-nantes.fr; 7 UMR INRA 1014 SECALIM, Oniris, route de Gachet, CS 40706, 44307 Nantes cedex 03, France; michel.federighi@oniris-nantes.fr

**Keywords:** wood surfaces, antimicrobial, survival, screening, hygiene, methods, properties

## Abstract

Some wood species have antimicrobial properties, making them a better choice over inert surfaces in certain circumstances. However, the organic and porous nature of wood raises questions regarding the use of this material in hygienically important places. Therefore, it is reasonable to investigate the microbial survival and the antimicrobial potential of wood via a variety of methods. Based on the available literature, this review classifies previously used methods into two broad categories: one category tests wood material by direct bacterial contact, and the other tests the action of molecules previously extracted from wood on bacteria and fungi. This article discusses the suitability of these methods to wood materials and exposes knowledge gaps that can be used to guide future research. This information is intended to help the researchers and field experts to select suitable methods for testing the hygienic safety and antimicrobial properties of wood materials.

## 1. Introduction

Wood is an organic material and a renewable resource of nature. It is an eco-friendly material as compared to glass, plastic, and metals that cause environmental disorders i.e., pollution or health hazards [1]. It is also an important constituent of nature-based themes aimed to improve the psychological well-being of inhabitants [2]. Untreated wood surfaces are traditionally used for food preparation, cutting, fermentation, and packaging [3]. Wood and wood products are also used as flooring and beddings in animal husbandry practices where they contribute to improvement in the health and welfare of animals [4,5]. Meanwhile, the safety of wood material in hygienically significant places is questioned, owing to its porosity and hygroscopic nature. However, studies have shown that some commonly used wood speices have antimicrobial activities [6,7,8] and can be looked on as a safe material for indoor uses in hygienically significant places [2,9] and as food contact surfaces [3,10,11]. Therefore, the antimicrobial properties of this material are investigated either to validate its safety as a hygienic surface or for the discovery and identification of the active antimicrobial compounds present in it [9,12,13,14,15,16].

Various diagnostic methods are used to determine the antimicrobial properties of wood to evaluate the safety of this material via screening tests and/or quantify the presence of any active compounds [6,16]. Moreover, such tests can help identify the factors affecting the antimicrobial behavior of wood such as the nature of the microbes (type and resistance), the wood characteristics and variability (age, location, part, and treatment) and the environment (humidity, moisture, and temperature) [7,8,10]. In addition, such methods can also be used to evaluate the efficacy of disinfectants and treatments used to increase the antimicrobial effectiveness of surfaces [17,18,19,20].

In general, antimicrobial properties of wood are studied via extractive-based methods, where compounds are extracted using solvents (S1 in Supplementary Materials) and then subjected to conventional antimicrobial testing methods such as agar diffusion and broth dilution [8,12,21,22]. Meanwhile, the direct methods such as surface contact test, microbe recovery protocols (S2 in Supplementary Materials), and bioluminescence assay can assess the surface contamination of wood [23,24,25]. However, to our knowledge, there are no specific standard methods available for wood material to directly determine its antimicrobial potential or surface contamination. Further, mass spectrometry and chromatography help in the identification and characterization of active compounds [16,26,27]. Each method has its own benefits and disadvantages regarding its suitability for the handler.

Few reviews exist on the subject of testing the antimicrobial potential of different materials [28,29,30,31]. It is believed that this is the first review of the suitability of these methods for wood and hygienically important microbes, particularly those that can be responsible for infections in the healthcare setting, and among them those being multiresistant to antibiotics. Therefore, this article aims to describe the available antimicrobial assays, their suitability to wood material in different forms, along with their advantages and disadvantages regarding utilization. This information is intended to serve as a guideline for researchers and field experts regarding the application of suitable methods in wood science, microbiology, hygiene, and the discovery of novel antimicrobial agents.

## 2. Literature Search Method 

**Figure 1:**
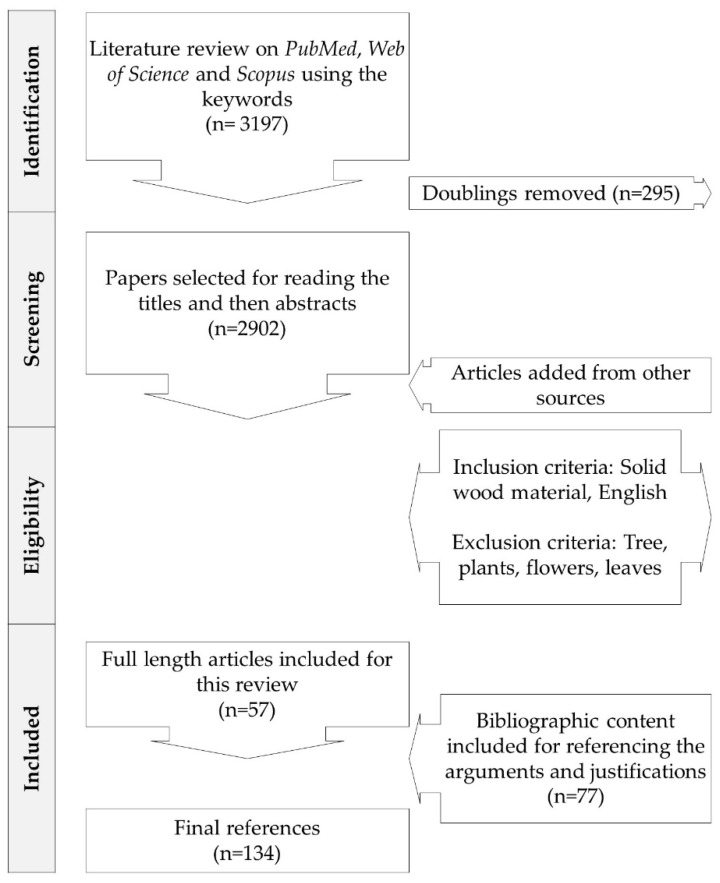
Flow chart of literature review methodology.

The literature was searched on *Scopus*, *PubMed*, and *The Web of Science* platforms as shown in Figure 1. The selected keywords were “Wood*” AND “antimicrobial” OR “antifungal” OR “antibacterial”. The timeline of research was set from the year 2000 to present (25/03/2020). The collected references were loaded to the Rayan^®^ platform for the screening of literature. The doublings were removed and the titles were read to scrutinize the suitable articles. Preference to inclusion was given to original research articles, published in the last 10 years, dealing with wood material, and written in English. Exclusion criteria were, non-wood material, conference presentations, posters, language other than English, and also the repeated similar methodologies reported by the same research group in multiple publications.

## 3. Results and discussion

A total of 57 articles were obtained to identify the methods of antimicrobial testing of wood material (Table 1). Further studies were added to describe the prospective methods (i.e., autobiography) of studying the antimicrobial properties of different compounds in the form of extractives.

**Figure 2:**
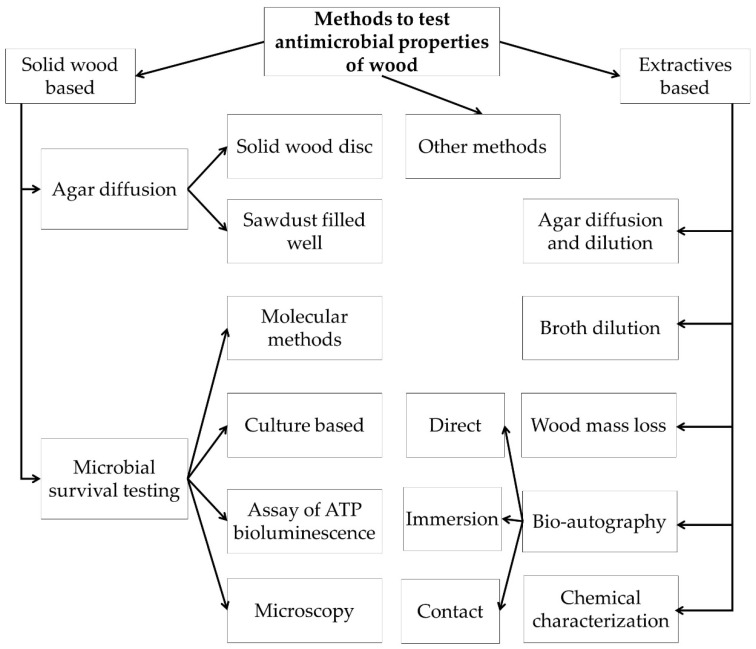
Flow diagram outlining review findings on the classification of methods to study the antimicrobial potential of wood material.

According to the literature findings, it was possible to categorize the methods into two broad groups based on form of test material used e.g., solid wood or extractives. Furthermore, they were subclassified into different groups according to the methodology, as shown in Figure 2.

### 3.1. Direct Methods

In this approach, the microbial survival after direct contact with wood samples is studied. These methods give a better understanding of the role of the physical structure of wood as a microorganism inhibitor. In general, they are easy to implement because usually no chemical handling or complicated preparation steps are required.

For research purposes, the direct methods may require an extra step of sterilization of the test material. Generally, the wood test samples are sterilized by autoclaving, ultraviolet irradiation, gamma radiation, fumigation, or by disinfection with alcohols [6,10,41,43,47,48,61,74]. It would be interesting to know if the sterilization methods interfere with the antimicrobial properties of wood material. For example, heat treatment may alter the chemical composition of the wood surfaces [21], and immersing wood pieces in ethanol may extract some compounds from them, thus, influencing the outcomes of the antimicrobial research.

#### 3.1.1. Agar Diffusion Method

The agar diffusion method is commonly used in routine for antibiotic susceptibility testing in clinical microbiology laboratories [75]. In this technique, an agar plate is conventionally used, and it is inoculated with a standardized bacterial or fungal suspension. The test sample, containing the potential active ingredients (added as a disc or deposited in a well created in the agar or a cylinder (plug)) is placed on the inoculated agar plate [76,77]. When such a system is incubated at a specific temperature, more often 37 °C, for a recommended time, the observation of growth inhibition around the test sample indicates the susceptibility of the incubated microbe [75]. This growth inhibition diameter is dependent on the antimicrobial susceptibility of an organism, the diffusion potential of testing antimicrobial agents in agar medium, and the efficacy of the active compounds [31,78].

The choice of the agar medium depends upon the type of microorganism being tested in the experiment. For many microbes, the recommendations have been defined by international organizations, such as the European Committee for Antimicrobial Susceptibility Testing [77] and the Clinical and Laboratory Standards Institute [76]. In general, the antimicrobial susceptibility of bacteria is tested on Mueller–Hinton agar [6]. However, plate count agar (PCA) [59], Iso-Sensitest^®^ agar [79], tryptone soy agar [13], and other nutrient mediums have also been used [80]. Antimycogram experiments generally involve the use of Sabouraud agar [79]. However, malt agar [59] and potato dextrose agar (PDA) are also employed for this purpose [66], depending upon the type of species being tested [81]. 

The incubation period depends on the growth requirement conditions of the tested microorganisms. Generally, most of the bacterial incubations vary from 18 to 24 h at 37 °C, while in case of fungi, 48 to 72 h are recommended at room temperature (25-30 °C) [45,72,82]. Then, the zone of inhibition (diameter) is measured to the nearest mm [71].

##### Direct Wood Disc Agar Diffusion Method (Antiboisgram)

**Figure 3:**
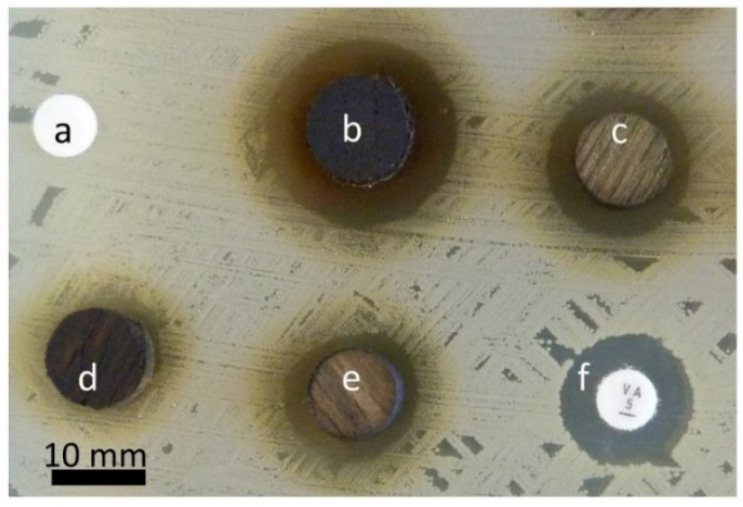
An antibiogram showing the results of filter paper discs (6 mm) and different oak tree wood discs (10 × 3 mm) tested against *Staphylococcus aureus* ATCC 29213 inoculated on a Mueller–Hinton agar plate: (**a**) negative control inert filter paper disc; (**b**) oak wood transversal cut; (**c**–**e**) oak wood longitudinal cut, and (**f**) positive control antibiotic (Vancomycin (Oxoid, Basingstoke, United Kingdom); ^©^Authors.

Munir et al. (2019) and Pailhoriès et al. (2017) reported a direct diffusion method to screen the bacterial growth inhibition potential of multiple wood species (Figure 3) [6,7]. In this method, a Mueller–Hinton agar plate was inoculated with a 0.5 McFarland bacterial suspension via swab streaking. Then, wood test samples with a disc form (2-4 mm thickness and 9-10 mm diameter) were directly placed on it. After an incubation time of 18-24 h, inhibition zones’ diameters were manually measured by two different readers. [7,8] used this method as a qualitative screening method and the presence of zone of inhibition was considered as a positive antimicrobial activity, while [6] further used this method and took into account the variability of the method for the interpretation of the results. This method can also be modified to test the antimicrobial properties and durability of treated solid wood samples (5 mm) against different fungi and bacteria [19,63,83,84]. Recently, a similar approach was applied by treating the *Melia azedarach* wood samples with acetone extract of *Withania somnifera* Fruit. Subsequently, the antimicrobial action was investigated against *Agrobacterium tumefaciens, Dickeya solani, Erwinia amylovora, Pseudomonas cichorii, Serratia pylumthica, Fusarium culmorum,* and *Rhizoctonia solani*. The positive antibacterial and antifungal responses were observed in the form of inhibition zones around samples on agar [34].

The direct diffusion method can give screening results very quickly and even the results of this technique can be interpreted in the absence of wood sterilization [7]. It can also help determine the influence of antimicrobial potential-affecting variables including the species of tree, part of tree [8], and geometry of cutting [6]. However, it is difficult to interpret in case of very low antimicrobial activity. In addition, the variability in this method can be high, making quantification a difficult task; therefore, uniform-sized test samples are recommended to overcome this difficulty [7].

##### Sawdust-Filled Well Diffusion Method 

**Figure 4:**
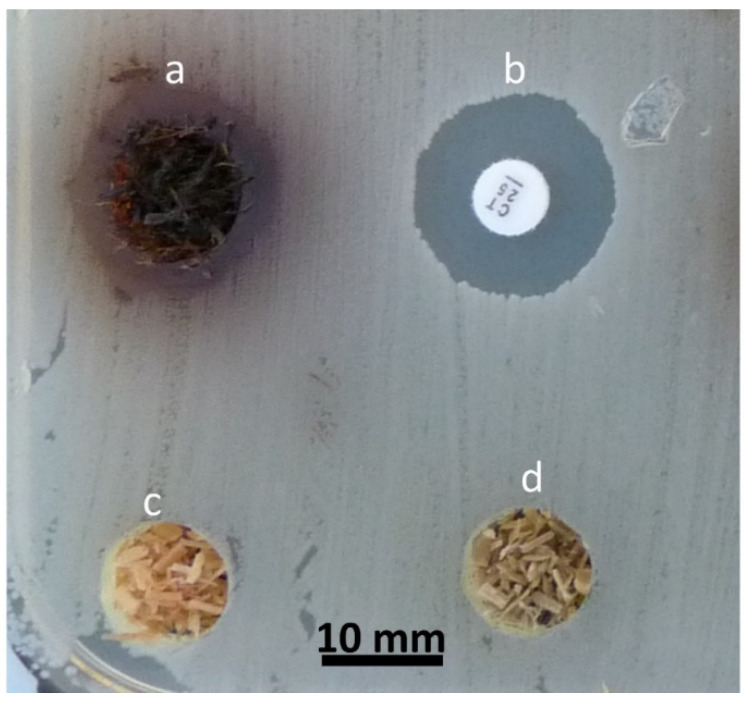
Antibiogram result of the well diffusion method to test the antimicrobial activity of sawdust (1–2 mm particle size), filled in wells (10 mm diameter) created in Mueller–Hinton agar against *Acinetobacter baumannii*: (**a**) oak wood showing the zone of inhibition around the well as a positive result; (**b**) positive control antibiotic disc (Colistin (Oxoid, Basingstoke, United Kingdom)–6mm diameter disc); (**c**) poplar sawdust with no activity, and (**d**) ash sawdust with no antimicrobial activity; ^©^Authors.

If the wood sample is only available in particulate and sawdust form, which is a common case in animal husbandry practices, then it can be placed in a well cut into the agar plate [4,5]. This method is a slight modification of agar diffusion method, where uniform-sized wells (5 to 10 mm) are punched aseptically with a sterile borer or a tip on agar plate [45]. Then, the sample particles are filled in these holes, and the system is incubated. The diameters of the zone of inhibition around these wells are measured as an indication of antimicrobial action [7] (Figure 4).

Although this method gives good results for screening purposes, it is not easy to fill the wells precisely without disrupting or contaminating the inoculated agar surface [7]; for example, Figure 4a shows that the few fibers are spreading out of the well. In addition, the particle size within samples may affect the diffusion and quantity of test material because finer particle sizes have a higher surface area to volume ratio compared to larger particles [85]. Therefore, granulometric studies are needed to standardize this protocol.

#### 3.1.2. Evaluation of Microbial Survival on Wood Surfaces

**Figure 5:**
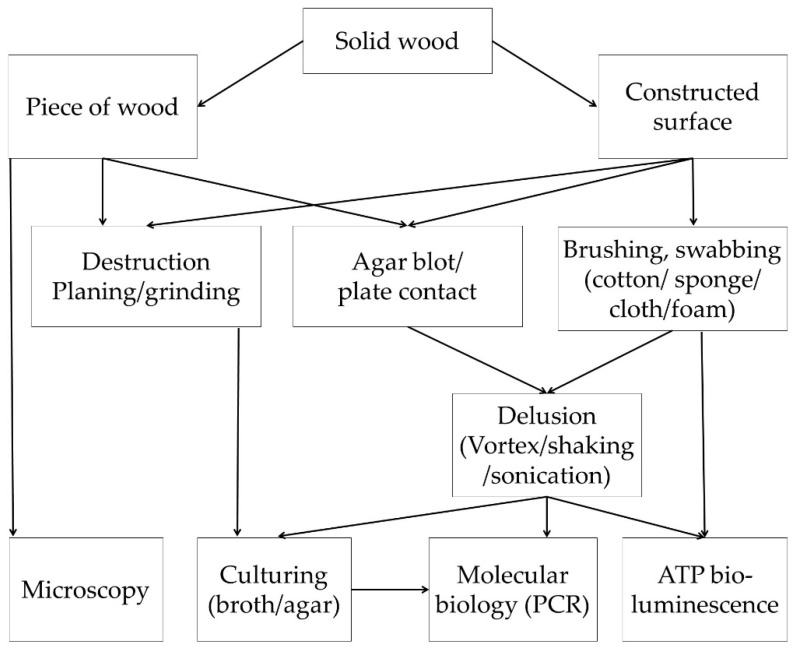
Flow diagram outlining review findings on the methods to study microbial survival on solid wood material.

The antimicrobial properties of wood can also be studied by observing the viability of microorganisms on wood. Recovery methods and visualization methods alone or in combination are employed to study the role of physical and chemical composition of wood to counter the microbial growth (Figure 5). Moreover, such methods also provide good evidence of safety studies of comparative materials such as plastic, glass, and steel.

In previous studies, recovery methods were described as destructive and non-destructive methods [52,86]; however, this classification may vary depdnding upon the availability of sample or employment of methodology. For example, planning can be both a destructive and non-destructive method for constructed surfaces. Therefore, Figure 5 describes more complete illustration of methodologies to study the microbial survival on wooden surfaces.

##### Microbial Recovery

Here, the recovery is defined as “*the percentage of cells detected from the number of initially inoculated cells on a surface*”. The microbial recovery gives information on their survival on different surfaces at different times [86]. Such methods are also used to study microbial adhesion and biofilm formation on wood surfaces [39]. In general, the microbial recovery from surfaces depends upon multiple factors, including the type of wood material, surface roughness, size of surface, porosity, moisture content, type of microbes, recovery method, contact time, skills of the handler, and the media used for collection, transport, and processing of samples [46,52,87].

As wood is a porous material with a very complex distribution of porosity [88], the recovery of total microbial content is difficult [86,89]. Even the transfer of microbes from the wooden contact surface to food is lower as compared to other surfaces [73]; for example, [10] reported that the transfer rates of *Listeria monocytogenes* from wood (0.55%) to cheese was lower than perforated plastics (1.09%) and glass (3%).

##### Culture-Based Methods 

A simple method of microbial recovery is blotting or agar plate contact, which involves directly touching the wood sample to agar to transfer microbes on it [2,17,18,23]. It involves contacting contaminated pieces of wood on agar at a specific pressure for a known time, e.g., 650 g for 10–20 s [60,90]. Kavian-Jaromi et al. studied the survival of *Klebsiella pneumoniae* and methicillin-resistant *Staphylococcus aureus* (MRSA) on Larch wood [*Larix decidua* (Mill)] [46]. Both heartwood and sapwood cubes (10 × 10 × 5 mm^3^) were inoculated with about 100 µL of bacterial suspension (10^6^ CFU ml^−1^). These samples were blotted onto blood agar plates (Columbia Blood Agar) after 0, 3, and 24 h of inoculation, and subsequently, the developed colonies were counted after 24 h of incubation at 37 °C. Gupta (2017) reported the contact RODAC (Replicate Organism Detection and Counting) plates method for the recovery of fungi and bacteria from different surfaces, including wood. These plates containing sterilized tryptic soy agar (TSA) and potato dextrose agar (PDA) for bacterial and fungal colonies respectively, were impressed upon test surfaces for 20 s and incubated directly at 37 °C and 24 °C for TSA and PDA plates, respectively. They also compared this method with a vacuuming and bulk rinsate method. Vacuuming was similar to air sampling for microbes with certain modifications adapted for surfaces. The contact method showed slightly higher recovery than vacuuming, and the bulk rinsate method gave 2 times higher recovery compared to the aforementioned methods.

Another direct method has been described in the literature where inoculated food contact surfaces, including wood [40], were covered with agar and after incubation, nitroblue tetrazolium solution (pale yellow) was used to stain colonies (purple) at the agar–test surface interface. Stained colonies could be readily detected and counted, and this method gave 5 times higher recovery than the swabbing method [91].

A simple rinsing of a wood surface with normal saline to collect microbes has also been reported [47,67]. However, this method is not very suitable for porous materials such as wood because microbes may descend in the depth of pores and do not come out with rinsing solution; in addition, even the surface-adhered microbes would not detach. Meanwhile, elusion-dependent methods recover the higher microbial concentrations from such surfaces [48,92]. They involve the direct immersion of contaminated pieces of test material (e.g., cubes, sawdust, shavings) or a collection device (e.g., swabs, sponges) in an eluent (sterilized phosphate buffer saline or peptone water) and then a physical dissociation method such as shaking, sonication, vortexing, or Stomacher used to recover the microorganisms [37,44,53,58,59,60,62,93,94]. Then, this suspension is further vortexed for 5–20 s and plated using serial dilution when appropriate [50]. Although this method gives higher recovery than the contact and vacuum method [92], the question arises if all the microbes are recovered from wood by this method. Earlier, Vainio-Kaila et al. [68] used a similar technique to remove all adhered *L. monocytogenes* and *Escerichia coli* cells from the surface of wood and glass samples. Samples were vortexed in 15 mL BHI (brain heart infusion) broth for 5 s. To enumerate the colony forming units (CFU), the suspension was subjected to a plate count method. Meanwhile, the test samples after microbial recovery were re-incubated in broth to determine remaining microbial quantity; however, no qualitative growth was observed after 24 h of incubation. This method has also been used to test the survival of microbes on wood shavings [53,56]. [62] used this method with certain modifications for the evaluation of antibacterial activity on grounded high-density polyethylene, expanded polystyrene, pine, and poplar wood. The materials were ground to obtain 0.4 g of each, and they were then suspended in 20 mL of buffered peptone water. The *Staphylococcus aureus* bacterial suspension was prepared and added in the same, together with test material, to obtain the final bacterial concentrations of around 1 × 10^5^–3 × 10^5^ CFU mL^−1^ adjusted by the McFarland turbidity method. Then, the suspension was vortexed to homogenize and incubated at 37 °C for 24 h. After the incubation time, the decimal dilutions of suspension were made in tryptone saline solution, and then the TEMPO^®^ system was used to quantify the remaining viable bacterial cells.

In addition, microbes are also collected by swabbing [20] and by destructive methods such as grinding [59] and the planing [51] of wood, and then they were further subjected to vortexing protocol for recovery [18,52].

Swabbing is also a common method for collecting microorganisms from wooden surfaces [2,20,38,41,51,55,60,95,96,97,98,99]. The swabs can be wet or dry and could be in form of cotton, foam, cloth, and sponge [35,40,73,100]. The microbial collection depends on the type of swabbing approach adapted [35,46]. Ahnrud et al. reported that the sonicating swab device that combines swabbing, sonication, and suction can recover a significantly (*p* < 0.05) higher number of *L. monocytogenes* cells from wooden cutting boards as compared to sponge, foam, and cotton swabbing [34].

Wood is intrinsically porous, which allows organic debris and bacteria to descend into the pores of wood unless a highly hydrophobic residue covers the surface [2,89]. It is highly likely that the porous structure of wood provides valleys and holes in which microbes are protected from any swabbing action [11]. In addition, a higher number of microbes were recovered by swabbing a longitudinally cut wood surface as compared to a transversally cut wooden surface owing to the difference of surface porosity [101,102].

In general, the recovery methods give lower recovery from wood in dry conditions as compared to moist surfaces [52]. Welker et al. reported that the recovery of *E. coli* with the sponge swab method was similar on plastic and moist maple wood, while it was very low on dry wood (0.1%) and plastic (0.25%) [73]. Imhof et al. reported that the recovery of *Listeria* spp. from spruce wood was higher by an abrasive (planing) method as compared to swabbing (cotton rolls) in dry conditions; however, both methods gave similar results when wet with a low detection sensitivity of < 32 CFU/cm^2^ [51]. The role of surface moisture is also linked to longer survival of microbes, which could lead to higher cultivable microbial recovery. Ismail et al. [52] reported that the microbial recovery rate from wood was greater at higher moisture contents, regardless of the method of recovery (palning, brushing, or grinding), wood species (pine or poplar), and microorganism (*E. coli, L. monocytogenes,* or *Penicillium expansum*). For example, the recovery rates for *E. coli* at 18% and 37% moisture contents were 19% and 30% from pine and 8% and 27% from poplar wood, respectively. They also reported that the grinding method was found to be the most sensitive, giving the highest recovery rates in all conditions as compared to planing and a brushing method. In another study, Coughenour et al. [38] reported that the addition of Bovine Serum Albumen to the glass, wood, vinyl, plastic, and cloth surfaces enabled methicillin-resistant *S. aureus* to survive for significantly longer duration (*p* < 0.001). Interestingly, the recovery of number of CFU was significantly lesser on surfaces stored in 45–55% versus 16% relative humidity.

##### Molecular Biology Methods 

The specific amplification of nucleic acids, such as in polymerase chain reaction (PCR), can be employed as a culture-independent method to investigate the microbial diversity in different environmental settings with complex mixture communities, non-cultivable viable cells (NCVC), interfering contaminants, and low levels of target DNA [103]. In first step of the PCR technique, the genetic material is isolated and purified from the target samples [104]. The step can also be a culture-independent method; for example, [32,33] used the swabbing of cutting boards for sample collection. Further, they vortexed the samples to obtain microbes and then extracted DNA without culturing these samples. Finally, they used the pyrosequencing technique to identify bacteria.

In PCR, the probes to target various genes can be designed depending upon the objective of study. The common probes are the phylogenetic probes to get information about the phylogeny of the microorganism, functional gene probes to identify the particular activity of the microbial community, and the species-specific primers to determine the presence of a specific microorganism [104]. These probes can also be used to detect the quantitative growth of microbes in different conditions. [56,57] studied the survival of fecal microbes in contact with wood material. For microbial recovery, the contaminated wood particles (3 g) were transferred to sterile plastic bags containing an extraction buffer (1:10 ratio). The samples were mechanically treated in a Stomacher lab blender for 3 min at 260 rpm to dislodge the adhering bacteria. The obtained suspension was used for DNA extraction and culturing for counting bacterial numbers. The decrease in the number of microbes as compared to initial inoculation was regarded as a loss of microbial survival in contact with wood material.

Genetic identification approaches are also important to recover NCVCs that are in a dormant state in the environment but are capable of cell division, metabolism, or gene transcription (mRNA production). Generally, the culture-based methods cannot identify NCVCs. [35] reported that the efficiency of sponge and swab recovery with culture-based methods, to obtain *Erwinia herbicola* from different laminated wood surfaces, was very low (11% and 29%) as compared to qPCR.

As the DNA of dead microbes can persist for an extended period in environments, the molecular assessment (especially for DNA-based methods) can overestimate the viable cell numbers [105]. There are other markers proposed to overcome this limitation. Messenger ribonucleic acid (mRNA) is turned over rapidly in living bacterial cells. It has very short half-life inside the cell and can be used as a marker for microbial viability and identification of NCVCs [104]. The nutritional stimulation of bacterial cells immediately produces a significant amount of rRNA precursors (pre-rRNA); these strands are easier to detect than mRNAs [103]. Therefore, they can also be used as a marker for differentiating NCVC from dead cells that have been inactivated by UV irradiation, pasteurization, serum exposure, and chlorine [105]. However, these techniques have not been used to study the microbial survival on wood, but the prospect has to be employed.

##### ATP Bioluminescence Assay

The ATP bioluminescence assay can rapidly detect the adenosine triphosphate (ATP), which is a component of all living cells. This process uses the luciferin enzyme derived from fireflies. When ATP from test samples reacts with luciferin in the presence of oxygen, the bioluminescence is generated as a byproduct, which is measured in relative light units (RLU) [24,106,107,108]. This device is generally applied on the surfaces after cleaning to detect the remaining contamination of microbes and organic matter in real time [73]. This method uses the swabbing of surfaces to collect organic matter, and results can be understated because of the lower recovery of microbes [106]. Shimoda et al. [25] used ATP assay to test the contamination of hospital surfaces (melamine, vinyl chloride, stainless steel, wood, and acrylonitrile–butadiene styrene) and found that wood material showed significantly high RLU values with huge variability. The authors cautioned that ATP values on wood surface were likely to be inaccurate because the CFU on all surfaces were same. Likewise, the sensitivity and specificity of a bioluminescence test as compared to the aerobic colony count method were reported to be 46% and 71% [108]. A recent study has also shown that ATP measurement is not an appropriate tool to measure bacterial contamination on wood and bamboo surfaces in hygienically important places [24]. These variations are linked to the organic nature of wood, and some traces of ATP may be present in this material, which interferes with the results, as [73] reported a higher level of bioluminescence in new wood samples as compared to plastic. From the results of these studies, it can be concluded that an initial reading before contamination and another after contamination can give clearer information about actual microbial presence. Moreover, ATP bioluminescence assay should be coupled with culture-based methods to determine the microbial survival on wood.

##### Microscopy of Microbes on Wood 

The microscopic approaches are promising tools to study the morphology and probes as an indication of microbial survival and viability on different surfaces. Scanning Electron Microscopy (SEM) is widely used to observe the presence of contaminants on wood surfaces. Many articles are found in the literature with biofilm structure analyses by SEM to describe the morphological effects of fungi or bacteria distribution [39,51,73,97,101,109,110,111,112,113,114]. Cruciata et al. [39] described the formation and characterization of early bacterial biofilms on different wood species (Calabrian chestnut, Sicilian chestnut, cedar, cherry, ash, walnut, black pine, and poplar woods) used in dairy production. By using SEM, they observed a visible exopolysaccharide matrix that is typical of biofilm structures and showed the presence of both rod and coccus bacteria on the wood surfaces.

However, SEM is restricted to 2D exploration, and 3D observation of microbial colonization inside the pores and cracks of wood is very difficult [115]. Furthermore, such a method requires a series of highly invasive fixation steps incompatible with live imaging and is unable to provide direct information on the survival status of bacteria on analyzed wood surfaces [116]. Moreover, direct microscopy such as environmental SEM can change the morphology of wooden structures and microbial cells during the imaging process [42,117]. Therefore, the application of a microscopy to study microbial survival and interaction with wood components is a challenging task.

**Figure 6:**
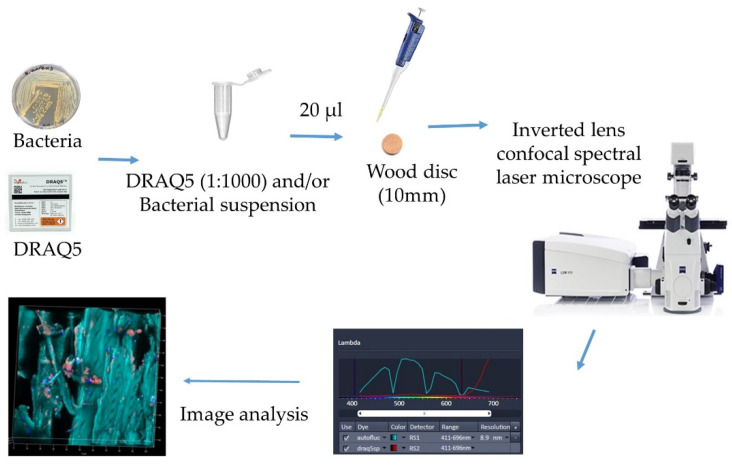
Methodology to observe DRAQ5-labeled bacteria with confocal spectral laser microscopy [adapted from Dubreil et al. [42]].

Confocal laser scanning microscopy (CLSM) in conjunction with digital image processing techniques has been reported as a potent non-invasive optical sectioning tool [115]. It allows micromorphologies of microbe interaction within wood to be examined at a depth around 50 µm of a specimen without incision, depending on the density of the wood sample [116]. Xiao et al. [116] reported that after fixation with glutaraldehyde, it was possible to locate fungal hyphae in wood, and counterstaining wood with fluorescent phospholipid probe enabled the visualization of bacterial colonization and even distinguished Gram types to detect them in wood cell walls. Dubreil et al. [42] developed an innovative method where they applied CSLM to observe *E.coli* labeled with a DNA probe DRAQ5 on poplar wood (Figure 6). This approach helped to visualize the presence and localization of bacterial cells, and it can be an interesting approach to determine the hygienic risk of microbial presence. However, this method did not give information of the viability of bacteria, and it even did not work well when applied on *S. aureus* bacteria. Recent work is being performed to optimize the visualization on hygienically important microbes on wood material by using spectral unmixing methods to analyze multilabeling and separate specifically fluorescence from bacteria, fluorescence from live/dead kit and the autofluorescence of wood (unpublished data).

### 3.2. Methods to Study the Antimicrobial Properties of Wood Extractives 

Wood contains biochemical compounds that enhance its resistance to microbial degradation. These special chemicals or extractives are not structural components, so they can be extracted by using different solvents [118,119]. The quantity and type of extractives vary between wood species even within different parts of wood in the same tree [119]. Moreover, the antimicrobial activities of different extractives in various plants vary according to solvents used [8]. On one side, extraction-based protocols give precise information of antimicrobial activity, and on the other side, the extraction adds an extra step in the antimicrobial test and requires chemical handling.

#### 3.2.1. Agar Diffusion and Dilution Methods

The antimicrobial properties of wood extractives can be tested by different agar diffusion-based methods that are classified based on loading the test solution on agar.

In the first method, wooden extractives in viscous form can be directly loaded on inoculated agar as circular points and after the incubation period, zones of inhibitions are observed as indicators of antimicrobial activity [22].

In the well method, extractives (50–100μL) are directly pipetted into 6 mm diameter wells made in the agar. First, the extractive solutions are diluted to different concentration [65,120,121].

**Figure 7:**
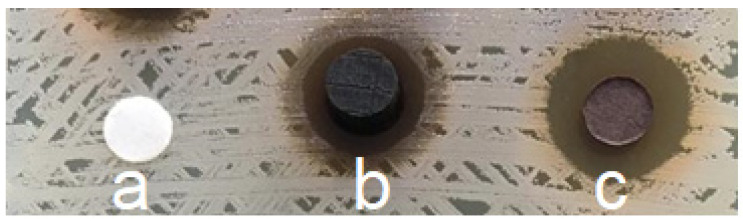
Antibiogram to test the antimicrobial properties of oak wood (*Quercus petraea*) against *Staphylococcus aureus* with the agar diffusion method: (**a**) an inert filter paper disc (negative control); (**b**) a wooden disc showing antimicrobial activity by forming a zone of inhibition and (**c**) a filter paper disc impregnated with wood extractives (10 mg extractive content extracted with methanol) showing antimicrobial activity by forming a zone of inhibition; ^©^Authors.

In the filter paper disc diffusion method, the extractives in different concentrations are impregnated into filter paper discs that are subsequently placed on agar plates. During the test disc preparation, the absorption potential of filter paper discs can vary depending upon the type of paper material being used. There are commercial paper discs available that have a diameter of 6 mm. Their general application is in antimicrobial sensitivity experiments in clinical microbiology laboratories. These discs are impregnated with 15–50 µL of stock solutions [122]. However, different sizes of the discs ranging from 5 to 10 mm can be created from blotting paper or simple filter paper (Whatman, no. 1 or 3) [14,82] and they can be impregnated with 10–200 µL of test solution extracted from wood material [8,80,84]. However, some studies have reported the soaking method in which the crude extracts were dissolved in TWEEN-20 solvent [to emulsify carrier oil in water [123]] and 10% stock solutions were prepared. The blotting paper discs (6 mm diameter) were soaked in various dilute solvent extracts and dried for 5 min to avoid the flow of extracts in the test media [66,124]. The following step is air drying, maintaining the sterility of material. The repetition of impregnation and drying can allow the loading of more liquid on discs. Finally, the sample loaded filter paper discs are subjected to the agar diffusion method to study the antimicrobial properties (Figure 7).

Another method of using agar microdilution has been described in the literature, which involves the dispersion of a test compound in molten agar and dispensing the mixture into a 96-well microplate in a small volume of 100 μL per well, which allows a rapid, easy, and economical preparation of samples as well as providing a uniform and stable dispersion without the separation of the oil–water phases, which occurs in methods with liquid medium [125].

The extractives in different quantities can also be mixed with agar before pouring into Petri dishes. Later, the bacteria are inoculated by steaking or spreading [48]. This method is also used for studying the antifungal response of wood extractives, and for this purpose, a piece of agar from a fungi-cultured plate is taken and placed on the extractive-infused petri dish. The size of the circular growth of fungi on agar gives a reading of fungal resistance against extractives [79].

#### 3.2.2. Broth Dilution Methods

**Figure 8:**
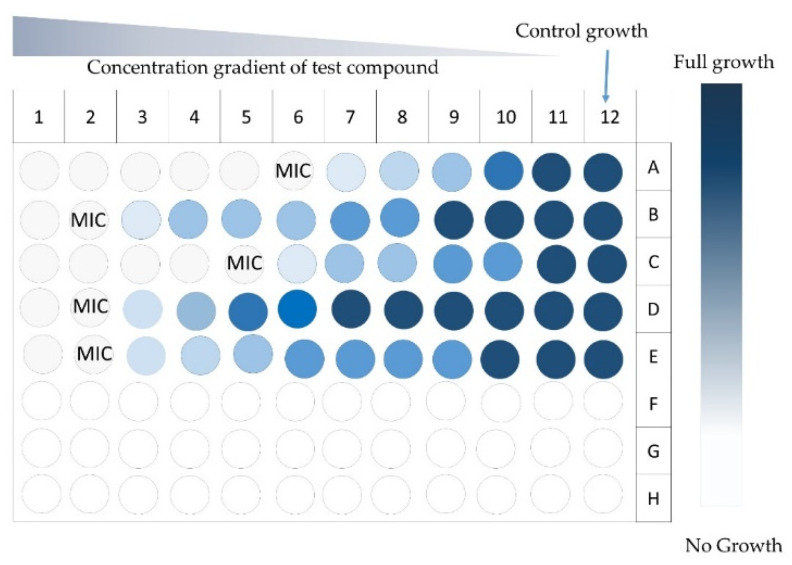
A 96-well plate showing results of the broth microdilution method for an antimicrobial test and minimum inhibitory concentration (MIC); ^©^Authors.

This method is more common to determine the minimum inhibitory concentration (MIC) [28], which is the lowest concentration of an antimicrobial product inhibiting the visible growth of a microorganism after overnight incubation [126]. It requires the homogenous dispersion of a sample agent in solvent, and dilutions of different concentrations are tested to determine MIC [31,64] (Figure 8).

If the purpose of an experiment is just to test the antimicrobial potential of wood extractives, only one selected dose can be added [71]. The inoculation, incubation, and reading can be performed manually or by an automated system, and the results can be read either by the formation of microbial colonies or the stoppage of growth [28,31]. In an automated method, the formation of bacterial colonies gives turbidity to the medium, and it is measured by spectrophotometry [71,75].

#### 3.2.3. Measurement of Wood Mass Loss to Decaying

Wooden surfaces are treated with a number of synthetic and natural products, including wooden extracts, to increase resistance against microbial biodegradation. Measurement of the loss of wooden mass to degradation over time is used as a parameter to evaluate the protective effect of surface treatment or wood itself. Cai et al. [36] studied the protective effect of *Pterocarpus* spp. extracts on Poplar samples against wood-degrading fungi. The wood was blast dried in an oven at 40 °C until the mass was constant and then immersed in the prepared extract solution for 2 h. The samples were dried again until the mass was constant. The control and treated samples were placed in culture flasks and incubated at 75% relative humidity and 28 °C for decaying for 3 months. Later, the samples were taken out, hyphae and impurities on the surface were removed, and the samples were oven-dried. The percentage of sample mass loss was used as an indication of the antimicrobial effect.

#### 3.2.4. Bioautography

This extractive-dependent method involves the hybridization of planar chromatography (for phytochemical analysis of extracts) with biological detection methods (for antimicrobial potential) [127]. The technique is similar to the agar diffusion method except that the tested compound diffuses from the chromatographic layer [14].

##### Direct Bioautography 

**Figure 9:**
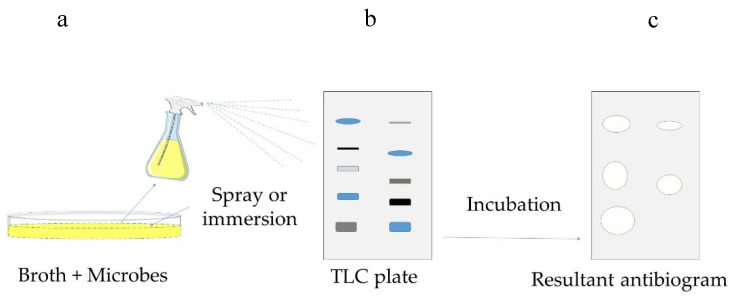
Schematic presentation of direct bioautographic method: (**a**) a developed chromatographic plate is placed in a dish; (**b**) agar is poured into this dish, and later, microbes are inoculated and (**c**) after the incubation time, the zones of inhibition can be seen on agar around the active antimicrobial compounds (the figure is adapted from [14,128,129,130]).

This is a widely used bioautographic method, which links detection on the adsorbent layer with biological tests performed directly on it [128]. In this method, extractive is loaded on a thin-layer chromatographic (TLC) plate to obtain a chromatogram. Further, this plate is dipped or sprayed with a suspension of microbes grown on a proper culture, and it is then incubated in a vapor chamber to provide a humid atmosphere [14,129,130]. In the case of anaerobic microbes, the scenario is different, the incubation in a sealed jar may result in high humidity potentially, causing a softening and peeling of silica gel layer from the aluminum base; the shorter incubation period and concentrated bacterial suspension are recommended to avoid this problem [131]. Finally, the inhibition of microbial growth can be spotted directly (Figure 9). To improve this visualization, [130] used p-iodonitrotetrazolium violet, which did not reduce the zone of inhibitions and was visible as white bands. The targeted compounds can also be identified using spectroscopic methods, mostly mass spectrometry, which can be performed directly on a TLC plate [27,128]. This high-throughput method enables analyses of many samples in parallel and the comparison of their activity, making both the screening and semi-quantitative analysis possible [128,130].

##### Contact Bioautography

**Figure 10:**
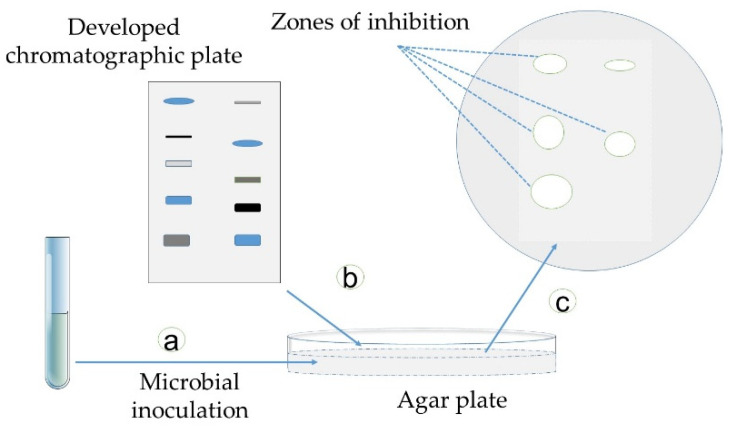
Schematic presentation of the contact bioautographic method: (**a**) microbes are inoculated on an agar plate; (**b**) a developed chromatographic plate is flipped over an agar plate to create a chromatographic image and transfer the active compounds, and inoculated plates are incubated for 48 h at 37 °C, and finally, (**c**) the zones of inhibition can be seen on the agar around the active antimicrobial compounds (adapted from [28,127,128,131]).

In this method, the TLC plate or paper chromatograms are placed in contact with the inoculated agar surface for some minutes or hours to allow diffusion [54]. Next, the plate is removed, and the agar layer is incubated for 1–3 days [26]. The zones of growth inhibition appear in the places where the antimicrobial compounds were in contact with the agar layer [131] (Figure 10). The visualization can be enhanced by using vital dyes [26].

##### Immersion (Agar-Overlay) Bioautography

**Figure 11:**
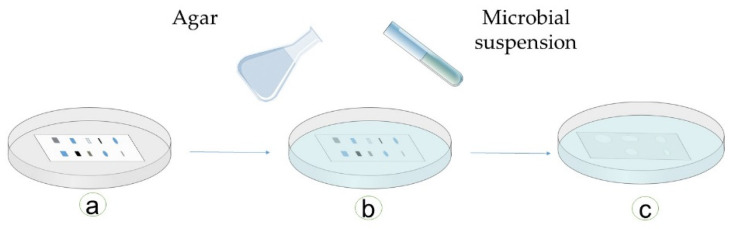
Schematic presentation of the immersion bioautographic method: (**a**) a developed chromatographic plate is placed in a dish; (**b**) agar is poured into this dish, and later, microbes are inoculated; (**c**) after an incubation time, the zones of inhibition can be seen on agar around the active antimicrobial compounds (adapted from [28,127,128,132]).

This is the combination of two formerly described methods. In this technique, an extractive inoculated, developed chromatographic plate is immersed in or covered with molten agar [127]. After the solidification of agar, the plate is seeded with the tested microorganisms and then incubated [132] (Figure 11).

#### 3.2.5. Active Antimicrobial Ingredient Identification

For the sake of active ingredient or compound identification, the wood extracts are fractioned by chromatographic and spectrophotometric techniques to obtain the pure compounds, which can be further tested for their antimicrobial properties by the conventional methods described above [49,65,133,134]. However, the fractioning of compounds to test for antimicrobial activities is a laborious bioactivity-guided isolation procedure, and it also yields an extremely low quantity of active substances after purification [12]. In this scenario, the characterized chemical profile can be labeled as antimicrobial compounds according to previous research done on them [16].

### 3.3. Other Methods 

There are several other ways to detect the antimicrobial properties of natural compounds, and they still remain to be tested for their application in wood science. One of such methods is inducing infection in animal models and using the dose of extractives as antimicrobial compounds to treat or eliminate the infection. In more sophisticated studies, the mode of action of different compounds is identified against different microorganisms. Plumed-Ferrer et al. [13] studied the antimicrobial effects of wood-associated polyphenols on food pathogens and spoilage organisms. They identified the mode of antimicrobial effect of these compounds by studying the microbial membrane permeability and membrane damage.

When it comes to bioaerosol quality of indoor air, the effect of the presence of wooden material on the microbial flora is also an important subject of research. Such studies need to utilize static chambers; however, there is no standard method published for wood material. There is an innovative study conducted by Vainio-Kaila et al. [70] regarding the effect of volatile organic compounds from *Pinus sylvestris* and *Picea abies* wood on *S. aureus*, *E. coli, Streptococcus pneumoniae,* and *S. enterica* Typhimurium. The experiment was carried out in a closed glass container (volume 1.9 L). First, 70 g of sawdust was placed on the bottom. A bacterial solution (20 μL) was inoculated on the glass discs on a rack above the bottom. After the incubation at room temperature for 2, 4, and 24 h, glass discs were dropped in test tubes to recover and enumerate the microbes by the plate count method. This method successfully measured the antimicrobial effect of volatile organic compounds on the microbial survival in different situations of time, air humidity, and sample moisture.

### 3.4. Pros and Cons of Mthods Used to Study Antimicrobial Behavior of Wood Material

A number of factors influence the choice of method selection to study the antimicrobial properties of wood materials. These factors are related to the availability of experimental material, test samples, purpose of study, and skills of handlers. The advantages and disadvantages of the methods discussed in this review are summarized in Table 2.

## 4. Conclusions 

This review summarizes the methods available studying the antimicrobial behavior of wood material. This information is intended to help field experts and researchers to find methods according to their needs and available resources.

Only a few publications were found using the direct diffusion method for screening the antimicrobial properties of solid wood material. However, this quick method shows the potential to be adapted as a standard screening protocol because of the direct nature of testing. It is noteworthy that a limit of this method is the lack of cut-off values for differentiating active and inactive materials, as such values are available for antibiotics. Therefore, further research is needed to apply this protocol, generate data to identify the variability of this method, and define criteria for interpreting the results of tests.

The literature showed that extractive-based methods are extensively used to identify the antimicrobial properties of wood and wood products. Broth dilution methods indicate the precise minimum inhibitory concentrations of extracted compounds with antimicrobial properties.

Direct bioautography shows good results for screening and the partial identification of active compounds responsible for the antimicrobial activity of wood. However, there is less information available regarding the particular use of contact and immersion bioautography application to search the antimicrobial behavior of wood material. Such studies can serve both purposes of active ingredient identification and their antimicrobial activity testing.

It is also evident that the recovery of microbes to study their survival on wood material remains a challenge. No standard protocol exists for such studies; hence, the methodology is adapted from the other comparative construction products. Consequently, the survival of pathogens on wooden surfaces may be misinterpreted. Future research should identify the recovery and/or survival of different microorganisms on wood regarding the variations related to wood species, physical condition, surface porosity, hygroscopicity, and roughness.

The use of genetic approaches such as quantitative PCR can enhance the efficiency of methods intended to study the microbial survival in contact with wood material. In addition, current microscopic approaches are not very successful to show the microbial survival on wooden surfaces. Therefore, future studies should address the question of microbial viability on wood by using metagenomics approaches and live/dead fluorescence microscopy.

## Figures and Tables

**Table 1:** Summary of publications selected for full-text review.

Material	Microorganism	Objective of the Study	Methods	Main Findings	Reference
Oak and pine	*Staphylococcus aureus, Salmonella enteritidis*	Survival of pathogens on wooden surfaces in healthcare facilities	Swabbing, planning, and plate count	Wood surfaces showed antimicrobial properties	[2]
Oak wood	Isolates of *S. aureus*	Oak in hospitals, the worst enemy of Staphylococcus aureus	Direct disc diffusion method	The method was efficient to show the antimicrobial properties of wood	[6]
Pine and spruce wood-associated polyphenols	*Salmonella, Listeria monocytogenes, S. epidermidis, S. aureus, Candida tropicalis, Saccharomyces cerevisiae*	The antimicrobial effects of wood-associated polyphenols on food pathogens and spoilage organisms	Microbial cell wall permeability and membrane damage	Several stilbenes showed antimicrobial activities against food pathogens and spoilage organisms	[13]
*Populus lasiocarpa, P. tomentosa*	*N/A*	Characteristics of antibacterial molecular activities in poplar wood extractives	GC/MS	The molecules were identified that are known to have antimicrobial properties	[16]
*Abies alba*, *Q. rubra*, European oak, *Fagus sylvatica*	*S.aureus, E. coli,* *P. aeruginosa, E. faecalis*	Direct screening method to assess antimicrobial behavior of untreated wood	Direct disc diffusion method	The method was efficient to show the antimicrobial properties of wood	[7]
Larch (*Larix decidua* Mill.) and Pine (*Pinus sylvestris* L.)	*Bacillus subtilis, S. aureus, Enterococccus faecium, Pseudomonas aeruginosa*	Testing the antimicrobial activities of different wood and their parts against different bacteria	Direct disc diffusion, paper disc diffusion	Antimicrobial activities depended upon the type of wood, part of tree, and type of bacteria	[8]
Spruce wood (*P. abies*), glass, polypropylene	*L. monocytogenes*	An assessment of bacterial transfer from wooden ripening shelves to cheeses	Food contact with surface	Wooden shelves had the lowest transfer rate of bacteria compared to other surfaces	[10]
Wood and other cutting boards	*S.* Enteritidis	Transfer of bacteria to food after cleaning the surfaces	Swabbing and contact press	Efficacy of cleaning methods was tested	[17]
Spruce wood shelves	*L. monocytogenes*	Survival of bacteria after the cleaning and sanitation of cheese preparation boards	Surface contact/blot planning and blending	Bacteria could not be cleaned by brushing and rubbing	[18]
Wood and other archeological objects	Variety of microbes	Isolation, characterization, and treatment of microbial agents responsible for the deterioration of archaeological objects	Swabbing	All samples were contaminated with various types of surface degrading microbes	[20]
*P. sylvestris, Picea abies*	*E.coli*	Effect of extractives and thermal modification on antibacterial properties	Plate count method	Thermal treatments and extraction influence on the antimicrobial properties of wood	[21]
*P. sylvestris, P. abies*	*S. aureus, E. faecalis, E. coli, Streptococcus pneumoniae*	Antibacterial properties of wooden extracts	Direct (extractive) agar diffusion method	Extractive showed antimicrobial properties	[22]
Oak and Douglas fir wood	Wood degrading microbes	Interaction of bacteria and fungi on wooden surfaces	Scanning electron microscopy and plate contact test	Environmental factors’ influence on the microbial interaction on wooden surfaces	[23]
Melamine, vinyl chloride, stainless steel, wood, and acrylonitrilebutadiene styrene	Total microbial count	ATP bioluminescence values are significantly different depending upon the material surface properties of the sampling location in hospitals	ATP bioluminescence, SEM, agar stamp/blotting	ATP and colony-forming unit (CFU) were different for wooden surfaces	[25]
Wood and plastic	Foodborne bacteria	Analysis of microbial community and food-borne bacteria on restaurant cutting boards	Pyrosequencing	Distribution of 32 genera was identified	[32,33]
Wood, plastic, vinyl, quarry clay tile	*L. monocytogenes*	Efficacy of sonicating swabs to recover microbes from surfaces	Sonicating swab compared to cotton, sponge, and foam swab	Sonicating swabs recovered significantly higher number of microbes	[34]
Contact surfaces including wood	*Erwinia herbicola*	Evaluation of two surface sampling methods for microbial detection on materials by culture and qPCR	Sponge and swabbing used for sample collection and tested by qPCR and plate count	qPCR is more sensitive than culturing, and swabbing was more efficient than sponge	[35]
*Pterocarpus* spp. and poplar wood	White and brown rot fungus	Evaluation of antimicrobial activity of ethanol and aqueous extracts	Wood mass loss calculation and gas chromatography-mass spectrometry	The wood extracts provided protection against degradation owing to antimicrobial properties	[36]
Wood and bamboo cutting boards	*Vibrio parahaemolyticus*	Efficacy of disinfectant to clean the cutting boards	Stirring method for microbial recovery	More microbes were recovered from plastic as compared to wood and bamboo	[37]
Wood cutting board and other surfaces	*Methicillin-resistant Staphylococcus aureus (MRSA)*	Microbial survival on five environmental surfaces	Swabbing	Survival and recovery of microbes depends upon the type of surfaces and moisture conditions	[38]
Calabrian and Sicilian chestnut, cedar, cherry, ash, walnut, black pine, poplar	*Salmonella, Listeria, E.cli, S. aureus,* Lactic acid bacteria (LAB)	Formation and characterization of early bacterial biofilms on different wood typologies	SEM for biofilm observation and paper disc method to determine antimicrobial activities	LAB represent efficient barriers to the adhesion of the main dairy, pathogens, probably due to their acidity and bacteriocin generation	[39]
Rubber wood cutting boards, plastic, glass	*E. coli, S. aureus*	Effectiveness of domestic antibacterial products in decontaminating food contact surfaces	Agar overlay method for microbial recovery	This method gave good results for testing the cleanability of surfaces	[40]
Pine and plastic	*E. coli, P. aeruginosa, S. aureus, L. monocytogenes*	Efficacy of electrolyzed water to inactivate different bacteria on cutting boards	Swabbing	Treatment was efficient for reducing microbial contamination	[41]
Poplar wood	*E.coli*	Confocal spectral microscopy—An innovative tool for the tracking of pathogen agents on contaminated wooden surfaces	Confocal spectral laser microscopy	The microbes could be located for their distribution by this method	[42]
*Melia azedarach* wood	*Agrobacterium tumefaciens, Dickeya solani, Erwinia amylovora, P. cichorii, Serratia pylumthica, Fusarium culmorum, Rhizoctonia solani*	Wood preservation potential of extracts	Direct diffusion method	Antimicrobial properties were observed using the disc diffusion method	[43]
Wooden toothpicks	Variety of microbes	Determination of microbial contamination of wood	Wet preparation techniques, concentration techniques, culture, biochemical tests	Wooden samples were found contaminated with a wide range of microorganisms	[44]
*Eucalyptus globulus* wood	*B. subtilis, S. aureus, S. epidermis, E. coli, C. krusei, P. aeruginosa C. parapsilosis, C. glabrata, C. albicans, Saccharomyces cerevisiae*	Extraction of bioactive compounds from biomass of forest management and wood processing	Well diffusion method	Antimicrobial compounds were identified	[45]
Spruce wood	*L. monocytogenes, L. innocua*	Comparison of methods for the detection of listeria on porous surfaces	Sponge swabbing	Porosity influences the recovery of microbes	[46]
Rubber wood and plastic	*L. monocytogenes*	Transmission of bacteria from raw chicken meat to cooked chicken meat through cutting boards	Rinsing with normal saline to remove bacteria and meat contact to study transmission	Surfaces play role in transmission of bacteria	[47]
Cork wood	*S. aureus* and *E. coli*	Evaluation of antimicrobial properties of cork	Agar dilution method	Cork has antimicrobial properties	[48]
Wood of *P. heldreichii* Christ. var. leucodermis	*S. aureus, S.epidermidis, E. coli, Enterobacter cloacae, Klebsiella pneumoniae, P. aeruginosa, C. albicans, C. tropicalis, C. glabrata*	Chemical composition and biological activity of the essential oil from pine wood	GC and GC/MS and Agar dilution method	Antimicrobial activities of pine wood were identified and characterized	[49]
Hardwood, carpets, vinyl and porcelain tiles	*S. aureus, Aspergillus niger*	Microbial survival on floor materials	Bulk rinsate, agar plate contact, vacuum suction	Microbial survival depends on the recovery method and surface type in hospitals (vet and human) and office buildings	[50]
Spruce fir boards (*P. abies*)	*L. monocytogenes, L. innocua*	Sanitizing wooden boards used for cheese maturation by means of a steam-mediated heating process	Planning and cotton swabbing and then stomacher	Both recovery methods showed identical results	[51]
Pine, poplar, spruce	*E. coli,* *L. monocytogenes, P. expansum*	Comparative study of 3 methods for recovering microorganisms from wooden surfaces in the food industry	Planning, grinding and brushing	Humidity, type of wood and microbe, and recovery method influenced the recovery rates	[52]
Sapwood and heartwood of the larch	*K. pneumoniae,MRSA*	Antimicrobial properties of wood against hygienic microbes	Blotting and vibration	Microbial quantities decreased after contact with wood	[53]
*Quercus baloot*	*C. albicans*	Evaluation of anticandidal potential of wood	Thin-layer chromatography, contact bioautography, disc diffusion method, broth microdilution	Chemical constituents were identified and antimicrobial activities were reported	[54]
Maple and Beech	Aerobic mesophilic microorganisms *Enterobacteriaceae, Pseudomonas spp.*	Hygienic aspects of using wooden and plastic cutting boards	Swabbing	Survival of microbes on different cutting boards before and after cleaning	[55]
Pine, larch, spruce, beech, maple, poplar, oak, polyethylene	*E.coli, E. faecium*	Studying the survival of pathogenic organisms in contact with wood material	PCR and culture-based recovery methods	Wood material has antimicrobial properties	[56,57]
Maple wood, steel, ceramic and carpet	*Enterobacter aerogenes*	Longer contact times increase cross-contamination of *Enterobacter aerogenes* from surfaces to food	Vortex for microbial recovery plate count method for enumeration	Contact time, food, and surface type all had highly significant effects on the log percent transfer of bacteria	[58]
Poplar	*E. coli, P. expansum*	Assessment of *Penicillium expansum* and *Escherichia coli* transfer from poplar crates to apples	Grinding/blending	There is a low transmission of microbes from wood to food (apple) as compared to glass and plastic	[59]
Wood, stainless steel, Formica, polypropylene	*Salmonella Typhimurium*	Recovery and transfer of Salmonella Typhimurium from four different domestic food contact surfaces	Swabbing (vortexting), contact pressing (635 g) and food contact	Number of microbes recovered and their transfer from wood to food was lowest compared to other surfaces	[60]
Poplar	*B. cereus spores, E. coli cells*	Behavior of bacteria on poplar wood crates by impedance measurements	Direct contact (wood in broth)	Microbes in contact with wood present in broth showed decrease in CFU	[61]
Poplar and pine	Total microbial counts, *S. aureus*	Hygienic properties exhibited by single-use wood and plastic packaging on the microbial stability for fish	Vortexing to recover microbes and enumerated by the TEMPO® system	Microbes decreased fastest on wood	[62]
*Leucaena leucocephala*	*Trichoderma viride, Fusarium subglutinans, A. niger*	Antimicrobial properties of wood treated with natural extracts	GC-MS, direct diffusion method	Antifungal properties were observed	[63]
*P. abies, Larix decidua*	*P. funiculosum, P. ochrochloron, A. niger, C.albicans, A. flavus, A. ochraceus, E.coli, S. aureus, Micrococcus flavus, B. cereus, L. monocytogenes, P. aeruginosa, Pectobacterium atrosepticum, Pec. carotovorum, Dickeya solani*	Antimicrobial properties of bark and wood extracts	GC-MS, microdilution method	The extracts showed antimicrobial properties, minimum inhibitory concentration (MIC) was determined	[64]
*Quercus incana*	*S.aureus, Micrococcus luteus, B. subtilis, E. coli, Ps. pickettii, Shigella flexneri, A. niger, A flavus*	Identification, isolation, and characterization of novel antimicrobial compounds	Disc diffusion method, well diffusion method	Two new compounds were identified with their antimicrobial properties	[65]
*Q. suber, Q. macrocarpa, Q. montana, Q. griffithii*, *Q. serrata*	*B. subtilis, S. pneumonia, E. coli, S. aureus, A. niger, Penicillium spp., Fusarium oxysporum*	Antimicrobial characterization combining spectrophotometric analysis of different oak species	Paper disc diffusion method and UV spectrophotometric analysis	Antimicrobial properties and active compounds were identified	[66]
Rubber wood	*Campylobacter jejuni*	Transfer of Campylobacter jejuni from raw to cooked chicken via wood and plastic cutting boards	Rinsing with normal saline and then counting CFU by combined most-probable-number (MPN)-PCR	Transfer during uncooked/cooked meat chopping on unscored and scored cutting boards	[67]
Heartwood of Scots pine (*P. sylvestris*)	*L. monocytogenes, E. coli*	Pine heartwood and glass surfaces: easy method to test the fate of bacterial contamination	Plate count and broth turbidity test	Wood does not allow the survival of microbes	[68]
*P. sylvestris* and *P. abies*	*MRSA, E.coli O157:H7*	Microbial survival on extractive-treated glass cylinders was studied	Vortexting and plate count method	Extractive showed antimicrobial properties	[69]
*P. sylvestris* and *P. abies*	*S. aureus, E. coli, S. pneumoniae, S. enterica Typhimurium*	Antimicrobial properties of volatile organic compounds (VOCs) of wood	Glass chamber and plate count method	VOCs reduced the microbial survival	[70]
30 species of trees	*B. cereus, S. aureus, L. monocytogenes, Lactobacillus plantarum, E. coli, Salmonella infantis, P. fluorescens, C albicans, Saccharomyces cerevisiae, A. fumigatus, Penicillium brevicompactum*	Antimicrobial and cytotoxic knotwood extracts and related pure compounds and their effects on food-associated microorganisms	Broth dilution and agar well dilution methods	Antimicrobial properties were observed	[71]
Beech wood (*F. sylvatica* L.)	*Gloeophyllum trabeum, Trametes versicolor*	Phenolic extractives of wound-associated wood of beech and their fungicidal effect	Spectrophotometrically analyzed and a paper disc screening test	Wood wounds have defensive chemicals to counter fungal invasion	[72]
Hard maple and plastic cutting boards	*E. coli*	Bacterial retention and cleanability of cutting boards with commercial food-service maintenance practices	Wet sponge swabbing	Microbial recovery was 0.25% and 0.1% from plastic and wood respectively in dry conditions and was similar in wet conditions	[73]

**Table 2:** Pros and cons of the methods used to study the antimicrobial behavior of wood material.

	Method Name	Procedure	Advantage	Disadvantage
Direct methods	Direct diffusion method (Well and disc)	The wood material is directly placed on microbe-inoculated agar or in a well and incubated for recommended time Presence of the zone of inhibition is considered a positive result	1. Rapid and time saving 2. Applicable for low amount of material 3. Adapted for screening	1. Disc preparation time 2. High variability for quantitative applications 3. Studies only the effect of agar-diffused chemicals 4. May require the sterilization of wood samples
	Culture-based microbial survival test	Initial microbial quantity is inoculated on wood samples and after the incubation time, the microbes are recovered, cultured, and viable cells are counted	1. Can study the structural and chemical role of wood components 2. Qualitative and quantitative results 3. Applicable for low amount of material	1. Difficulty in recovering all microbes present in pores 2. Microbial quantification is an extra step needed 3. Only viable cells are identified, while there can be still non-viable infectious cells present
	Microscopy	The behavior and distribution of inoculated microbes on wooden structures is observed via microscopy	1. Rapid and time saving 2. Applicable for low amount of material 3. Adapted for screening	1. May require the fixation of samples 2. Difficult to differentiate microbial structures from wooden structures 3. May require competencies of image analysis
	ATP luminescence	The ATP of microbes on wood is measured	1. Rapid and easy 2. Applicable for low amount of material 3. Adapted for screening	1. Difficult to differentiate the microbial ATP from other organic debris 2. Adapted only for solid surfaces
	Molecular biology methods	The quantity and viability of microbes is tested via nucleic acid amplification	Accurately measures the microbial survival	1. Expensive 2. Require sophisticated handling
Extractive based methods	Extractive-based diffusion and dilution method	Extractives are placed on agar or in agar wells, or in broth, after loading on filter paper discs or directly	1. Adapted for qualitative and quantitative antimicrobial studies 2. Specific chemicals can be extracted depending upon the solvent used	1. Involves chemical handling Extra step of extraction 2. One solvent cannot extract all active components 3. Does not study the role of structure of wood
	Bioautography	Extractives are loaded on a chromatographic layer, and then the diffusion of active chemicals is studied for their antimicrobial properties	1. Adapted for qualitative antimicrobial studies 2. Specific chemicals can be extracted depending upon the solvent used and identified on the basis of their diffusion on the chromatographic layer	1. Involves chemical handling and extraction 2. One solvent cannot extract all active components 3. Does not study the role of structure of wood 4. Not a quantitative method
	Mass spectrometry	The total profile of microbes is measured	1. Applicable for a low amount of material 2. Accurately measure the content of the active ingredient	For more specific results, the identified compounds are supposed to be tested by other culture-based methods

## Supplementary Materials

The following are available online at https://www.mdpi.com/2079-6382/9/5/225/s1.

## References

[1] Hill C.A.S., Dibdiakova J. (2016). The environmental impact of wood compared to other building materials. Int. Wood Prod. J..

[2] Kotradyova V., Vavrinsky E., Kalinakova B., Petro D., Jansakova K., Boles M., Svobodova H. (2019). Wood and its impact on humans and environment quality in health care facilities. Int. J. Environ. Res. Public Health.

[3] Aviat F., Gerhards C., Rodriguez-Jerez J., Michel V., Bayon I.L., Ismail R., Federighi M. (2016). Microbial Safety of Wood in Contact with Food: A Review. Compr. Rev. Food Sci. Food Saf..

[4] Munir M.T., Belloncle C., Irle M., Federighi M. (2019). Wood-based bedding in poultry production: A review. World’s Poult. Sci. J..

[5] Munir M.T., Irle M., Belloncle C., Federighi M. (2019). Wood Based Bedding Material in Animal Production: A Minireview. APDV.

[6] Pailhories H., Munir M.T., Aviat F., Federighi M., Belloncle C., Eveillard M. (2017). Oak in Hospitals, the Worst Enemy of Staphylococcus aureus. Infect. Cont. Amp. Hosp. Epidemiol..

[7] Munir M.T., Aviat F., Pailhories H., Eveillard M., Irle M., Federighi M., Belloncle C. (2019). Direct screening method to assess antimicrobial behavior of untreated wood. Eur. J. Wood Wood Prod..

[8] Laireiter C.M., Schnabel T., Köck A., Stalzer P., Petutschnigg A., Oostingh G.J., Hell M. (2013). Active Anti-Microbial Effects of Larch and Pine Wood on Four Bacterial Strains. BioResources.

[9] Munir M.T., Pailhories H., Eveillard M., Aviat F., Lepelletier D., Belloncle C., Federighi M. (2019). Antimicrobial Characteristics of Untreated Wood: Towards a Hygienic Environment. Health.

[10] Ismail R., Aviat F., Gay-Perret P., Le Bayon I., Federighi M., Michel V. (2017). An assessment of L. monocytogenes transfer from wooden ripening shelves to cheeses: Comparison with glass and plastic surfaces. Food Control..

[11] Filip S., Fink R., Oder M., Jevšnik M. (2012). Hygienic acceptance of wood in food industry. Wood Sci. Tech..

[12] Abedini A., Colin M., Hubert J., Charpentier E., Angelis A., Bounasri H., Bertaux B., Kotland A., Reffuveille F., Nuzillard J.-M. (2020). Abundant Extractable Metabolites from Temperate Tree Barks: The Specific Antimicrobial Activity of Prunus Avium Extracts. Antibiotics.

[13] Plumed-Ferrer C., Väkeväinen K., Komulainen H., Rautiainen M., Smeds A., Raitanen J.-E., Eklund P., Willför S., Alakomi H.-L., Saarela M. (2013). The antimicrobial effects of wood-associated polyphenols on food pathogens and spoilage organisms. Int. J. Food Microb..

[14] Valgas C., de Souza S.M., Smânia E.F.A., Smânia A. (2007). Screening methods to determine antibacterial activity of natural products. Brazi. J. Microb..

[15] Smailagić A., Ristivojević P., Dimkić I., Pavlović T., Dabić Zagorac D., Veljović S., Fotirić Akšić M., Meland M., Natić M. (2020). Radical Scavenging and Antimicrobial Properties of Polyphenol Rich Waste Wood Extracts. Foods.

[16] Peng W., Li D., Zhang M., Ge S., Mo B., Li S., Ohkoshi M. (2017). Characteristics of antibacterial molecular activities in poplar wood extractives. Saudi J. Biol. Sci..

[17] Soares V.M., Pereira J.G., Viana C., Izidoro T.B., Bersot L. (2012). dos S.; Pinto, J.P. de A.N. Transfer of Salmonella Enteritidis to four types of surfaces after cleaning procedures and cross-contamination to tomatoes. Food Microb..

[18] Zangerl P., Matlschweiger C., Dillinger K., Eliskases-Lechner F. (2010). Survival of Listeria monocytogenes after cleaning and sanitation of wooden shelves used for cheese ripening. Eur. J. Wood Wood Prod..

[19] Salem M.Z.M., Zidan Y.E., Mansour M.M.A., El Hadidi N.M.N., Abo Elgat W.A.A. (2016). Antifungal activities of two essential oils used in the treatment of three commercial woods deteriorated by five common mold fungi. Int. Biodeterior. Biodegrad..

[20] Elserogy A., Kanan G., Hussein E., Khreis S.A. (2016). Isolation, characterization and treatment of microbial agents responsible for the deterioration of archaeological objects in three jordanian museums. Mediter. Archaeol. Archaeom..

[21] Vainio-Kaila T., Rautkari L., Nordström K., Närhi M., Natri O., Kairi M. (2013). Effect of extractives and thermal modification on antibacterial properties of Scots pine and Norway spruce. Int. Wood. Prod. J..

[22] Vainio-Kaila T., Kyyhkynen A., Rautkari L., Siitonen A. (2015). Antibacterial Effects of Extracts of Pinus sylvestris and Picea abies against Staphylococcus aureus, Enterococcus faecalis, Escherichia coli, and Streptococcus pneumoniae. BioResources.

[23] Buchner J., Irle M., Belloncle C., Michaud F., Macchioni N. (2019). Fungal and bacterial colonies growing on weathered wood surfaces. Wood Mat. Sci. Eng..

[24] Lane K. (2019). The Efficacy of ATP Monitoring Devices at Measuring Organic Matter on Postharvest Surfaces. Master‘s Thesis.

[25] Shimoda T., Yano R., Nakamura S., Yoshida M., Matsuo J., Yoshimura S., Yamaguchi H. (2015). ATP bioluminescence values are significantly different depending upon material surface properties of the sampling location in hospitals. BMC Res. Notes.

[26] Moricz A.M., Ott P.G. (2017). Screening and Characterization of Antimicrobial Components of Natural Products Using Planar Chromatography Coupled with Direct Bioautography, Spectroscopy and Mass Spectrometry: A Review. Curr. Org. Chem..

[27] Moricz Á.M., Häbe T.T., Böszörményi A., Ott P.G., Morlock G.E. (2015). Tracking and identification of antibacterial components in the essential oil of Tanacetum vulgare L. by the combination of high-performance thin-layer chromatography with direct bioautography and mass spectrometry. J. Chromatogr. A.

[28] Balouiri M., Sadiki M., Ibnsouda S.K. (2016). Methods for in vitro evaluating antimicrobial activity: A review. J. Pharmaceuti. Anal..

[29] Cowan M.M. (1999). Plant Products as Antimicrobial Agents. Clin. Microbiol. Rev..

[30] Ncube N.S., Afolayan A.J., Okoh A.I. (2008). Assessment techniques of antimicrobial properties of natural compounds of plant origin: Current methods and future trends. Afr. J. Biotech..

[31] Rios J.L., Recio M.C., Villar A. (1988). Screening methods for natural products with antimicrobial activity: A review of the literature. J. Ethnopharmacol..

[32] Abdul-Mutalib N.-A., Amin Nordin S., Osman M., Ishida N., Tashiro K., Sakai K., Tashiro Y., Maeda T., Shirai Y. (2015). Pyrosequencing analysis of microbial community and food-borne bacteria on restaurant cutting boards collected in Seri Kembangan, Malaysia, and their correlation with grades of food premises. Int. J. Food Microb..

[33] Abdul-Mutalib N.-A., Nordin S.A., Osman M., Roslan A.M., Ishida N., Sakai K., Tashiro Y., Tashiro K., Maeda T., Shirai Y. (2016). The prevalence of foodborne pathogenic bacteria on cutting boards and their ecological correlation with background biota. Microbiology.

[34] Ahnrud G.P., Mendoza A.J., Hurley M.J., Marek P.J. (2018). Efficacy of a Sonicating Swab for Removal and Capture of Microorganisms from Experimental and Natural Contaminated Surfaces. Appl. Environ. Microbiol..

[35] Buttner M.P., Cruz P., Stetzenbach L.D., Cronin T. (2007). Evaluation of Two Surface Sampling Methods for Detection of Erwinia herbicola on a Variety of Materials by Culture and Quantitative PCR. Appl. Environ. Microbiol..

[36] Cai M., Lv H., Cao C., Zhang L., Cao R., Xu B. (2019). Evaluation of antimicrobial activity of Pterocarpus extracts. Ind. Crops Prod..

[37] Chiu T.-H., Duan J., Liu C., Su Y.-C. (2006). Efficacy of electrolysed oxidizing water in inactivating Vibrio parahaemolyticus on kitchen cutting boards and food contact surfaces. Lett. Appl. Microb..

[38] Coughenour C., Stevens V., Stetzenbach L.D. (2011). An Evaluation of Methicillin-Resistant Staphylococcus aureus Survival on Five Environmental Surfaces. Microb. Drug Resist..

[39] Cruciata M., Gaglio R., Scatassa M.L., Sala G., Cardamone C., Palmeri M., Moschetti G., Mantia T.L., Settanni L. (2018). Formation and Characterization of Early Bacterial Biofilms on Different Wood Typologies Applied in Dairy Production. Appl. Environ. Microbiol..

[40] DeVere E., Purchase D. (2007). Effectiveness of domestic antibacterial products in decontaminating food contact surfaces. Food Microb..

[41] Deza M.A., Araujo M., Garrido M.J. (2007). Efficacy of Neutral Electrolyzed Water To Inactivate Escherichia coli, Listeria monocytogenes, Pseudomonas aeruginosa, and Staphylococcus aureus on Plastic and Wooden Kitchen Cutting Boards. J. Food Protect..

[42] Dubreil L., Aviat F., Anthoine V., Ismail R., Rossero A., Federighi M. (2018). Confocal spectral microscopy—an innovative tool for tracking of pathogen agents on contaminated wooden surfaces. Eur. J. Wood Wood Prod..

[43] El-Hefny M., Salem M.Z.M., Behiry S.I., Ali H.M. (2020). The Potential Antibacterial and Antifungal Activities of Wood Treated with Withania somnifera Fruit Extract, and the Phenolic, Caffeine, and Flavonoid Composition of the Extract According to HPLC. Processes.

[44] Elom M.O., Ugah U.I., Omote V. (2014). Microbial Contaminants of Wooden Toothpicks in Abakaliki Metropolis, Ebonyi State, Nigeria. World J. Life Sci. Med. Res..

[45] Fernández-Agulló A., Freire M.S., González-Álvarez J. (2015). Effect of the extraction technique on the recovery of bioactive compounds from eucalyptus (Eucalyptus globulus) wood industrial wastes. Ind. Crops Prod..

[46] Frontino G. (2019). Comparison of Methods for Detection of Listeria on Wooden Shelves used for Cheese Aging: Challenges Associated with Sampling Porous Surfaces. Master’s Thesis.

[47] Goh S.G., Leili A.-H., Kuan C.H., Loo Y.Y., Lye Y.L., Chang W.S., Soopna P., Najwa M.S., Tang J.Y.H., Yaya R. (2014). Transmission of Listeria monocytogenes from raw chicken meat to cooked chicken meat through cutting boards. Food Control..

[48] Gonçalves F., Correia P., Silva S.P., Almeida-Aguiar C. (2016). Evaluation of antimicrobial properties of cork. FEMS Microbiol. Lett..

[49] Graikou K., Gortzi O., Mantanis G., Chinou I. (2012). Chemical composition and biological activity of the essential oil from the wood of Pinus heldreichii Christ. var. leucodermis. Eur. J. Wood Prod..

[50] Gupta M., Bisesi M., Lee J. (2017). Comparison of survivability of Staphylococcus aureus and spores of Aspergillus niger on commonly used floor materials. Am. J. Infect. Control.

[51] Imhof R., Schwendimann L., Scettrini P.R. (2017). Sanitising wooden boards used for cheese maturation by means of a steam-mediated heating process. J. Consum. Prot. Food Saf..

[52] Ismail R., Bayon I.L., Michel V., Jequel M., Kutnik M., Aviat F., Fédérighi M. (2015). Comparative Study of Three Methods for Recovering Microorganisms from Wooden Surfaces in the Food Industry. Food Anal. Methods.

[53] Kavian-Jahromi N., Schagerl L., Dürschmied B., Enzinger S., Schnabl C., Schnabel T., Petutschnigg A. (2015). Comparison of the antibacterial effects of sapwood and heartwood of the larch tree focusing on the use in hygiene sensitive areas. Eur. J. Wood Prod..

[54] Khurram M. (2011). Evaluation of anticandidal potential of Quercus baloot Griff. using contact bioautography technique. Afr. J. Pharm. Pharmacol..

[55] Lucke F.-K., Skowyrska A. (2015). Hygienic aspects of using wooden and plastic cutting boards, assessed in laboratory and small gastronomy units. J. Verbr. Lebensm..

[56] Milling A., Kehr R., Wulf A., Smalla K. (2005). Survival of bacteria on wood and plastic particles: Dependence on wood species and environmental conditions. Holzforschung.

[57] Milling A., Smalla K., Kehr R., Wulf A. (2005). Wulf The use of wood in practice–A hygienic risk?. Holz Roh Werkst..

[58] Miranda R.C., Schaffner D.W. (2016). Longer Contact Times Increase Cross-Contamination of Enterobacter aerogenes from Surfaces to Food. Appl. Environ. Microbiol..

[59] Montibus M., Ismaïl R., Michel V., Federighi M., Aviat F., Le Bayon I. (2016). Assessment of Penicillium expansum and Escherichia coli transfer from poplar crates to apples. Food Control..

[60] Moore G., Blair I.S., McDowell D.A. (2007). Recovery and transfer of Salmonella Typhimurium from four different domestic food contact surfaces. J. Food Prot..

[61] Revol-Junelles A.-M., Miguindou-Mabiala R., Roger-Maigné D., Millière J.-B. (2005). Behavior of Escherichia coli cells and Bacillus cereus spores on poplar wood crates by impedance measurements. J. Food Prot..

[62] Ripolles-Avila C., Hascoët A.S., Ríos-Castillo A.G., Rodríguez-Jerez J.J. (2019). Hygienic properties exhibited by single-use wood and plastic packaging on the microbial stability for fish. LWT.

[63] Salem M.Z.M., Mansour M.M.A., Elansary H.O. (2019). Evaluation of the effect of inner and outer bark extracts of sugar maple (*Acer saccharum* var. *saccharum*) in combination with citric acid against the growth of three common molds. J. Wood Chem. Tech..

[64] Salem M.Z.M., Elansary H.O., Elkelish A.A., Zeidler A., Ali H.M., EL-Hefny M., Yessoufou K. (2016). In vitro Bioactivity and Antimicrobial Activity of Picea abies and Larix decidua Wood and Bark Extracts. BioResources.

[65] Sarwar R., Farooq U., Naz S., Riaz N., Majid Bukhari S., Rauf A., Mabkhot Y.N., Al-Showiman S.S. (2018). Isolation and Characterization of Two New Antimicrobial Acids from Quercus incana (Bluejack Oak). BioMed Res. Int..

[66] Subhashini S., Begum S.M., Rajesh G. (2016). Antimicrobial characterisation combining spectrophotometric analysis of different oak species. Int. J. Herb. Med..

[67] Tang J.Y.H., Nishibuchi M., Nakaguchi Y., Ghazali F.M., Saleha A.A., Son R. (2011). Transfer of Campylobacter jejuni from raw to cooked chicken via wood and plastic cutting boards. Lett. Appl. Microb..

[68] Vainio-Kaila T., Kyyhkynen A., Viitaniemi P., Siitonen A. (2011). Pine heartwood and glass surfaces: Easy method to test the fate of bacterial contamination. Eur. J. Wood Wood Prod..

[69] Vainio-Kaila T., Zhang X., Hänninen T., Kyyhkynen A., Johansson L.-S., Willför S., Österberg M., Siitonen A., Rautkari L. (2017). Antibacterial Effects of Wood Structural Components and Extractives from Pinus sylvestris and Picea abies on Methicillin-Resistant Staphylococcus aureus and Escherichia coli O157:H7. BioResources.

[70] Vainio-Kaila T., Hänninen T., Kyyhkynen A., Ohlmeyer M., Siitonen A., Rautkari L. (2017). Effect of volatile organic compounds from Pinus sylvestris and Picea abies on Staphylococcus aureus, Escherichia coli, Streptococcus pneumoniae and Salmonella enterica serovar Typhimurium. Holzforschung.

[71] Valimaa A.-L., Honkalampi-Hämäläinen U., Pietarinen S., Willför S., Holmbom B., von Wright A. (2007). Antimicrobial and cytotoxic knotwood extracts and related pure compounds and their effects on food-associated microorganisms. Int. J. Food Microb..

[72] Vek V., Oven P., Humar M. (2013). Phenolic extractives of wound-associated wood of beech and their fungicidal effect. Int. Biodeteriorat. Biodegradat..

[73] Welker C., Faiola N., Davis S., Maffatore I., Batt C.A. (1997). Bacterial Retention and Cleanability of Plastic and Wood Cutting Boards with Commercial Food Service Maintenance Practices. J. Food Prot..

[74] Nikiforuk A.M., Cutts T.A., Theriault S.S., Cook B.W.M. (2017). Challenge of Liquid Stressed Protective Materials and Environmental Persistence of Ebola Virus. Sci. Rep..

[75] Bonnet R., Kahlmeter G., Leclercq R., Cornaglia G., Courcol R., Herrmann J.-L. (2012). Antimicrobial susceptibility testing. European Manual of Clinical Microbiology.

[76] CLSI Performance Standards for Antimicrobial Disk Susceptibility Tests, 13th ed. https://clsi.org/standards/products/microbiology/documents/m02/.

[77] EUCAST EUCAST Disk Diffusion Method for Antimicrobial Susceptibility Testing, Version 8.0. https://www.eucast.org/documents/publications_in_journals/.

[78] Jorgensen J.H., Ferraro M.J. (2009). Antimicrobial Susceptibility Testing: A Review of General Principles and Contemporary Practices. Clin. Infect. Dis..

[79] Khan M.N., Ngassapa O., Matee M.I.N. (2000). Antimicrobial Activity of Tanzanian Chewing Sticks Against Oral Pathogenic Microbes. Pharm. Biol..

[80] Nakmee P.S., Khuntong S., Nuengchamnong N. (2016). Phytochemical Constituents with Antimicrobial and Antioxidant Activities from Xylia xylocarpa (Roxb.) Taub. Sawdust Extracts. Chiang Mai J. Sci..

[81] Kim S.W., Kim K.S., Lamsal K., Kim Y.-J., Kim S.B., Jung M., Sim S.-J., Kim H.-S., Chang S.-J., Kim J.K. (2009). An in vitro study of the antifungal effect of silver nanoparticles on oak wilt pathogen Raffaelea sp.. J. Microbiol. Biotechnol..

[82] Das K., Tiwari R.K.S., Shrivastava D.K. (2010). Techniques for evaluation of medicinal plant products as antimicrobial agents: Current methods and future trends. JMPR.

[83] Mansour M.M.A., Salem M.Z.M. (2015). Evaluation of wood treated with some natural extracts and Paraloid B-72 against the fungus Trichoderma harzianum: Wood elemental composition, in-vitro and application evidence. Int. Biodeterior. Biodegrad..

[84] Salem M.Z.M., Zidan Y.E., El Hadidi N.M.N., Mansour M.M.A., Abo Elgat W.A.A. (2016). Evaluation of usage three natural extracts applied to three commercial wood species against five common molds. Int. Biodeterior. Biodegrad..

[85] Lu H., Ip E., Scott J., Foster P., Vickers M., Baxter L.L. (2010). Effects of particle shape and size on devolatilization of biomass particle. Fuel.

[86] Ismail R., Aviat F., Michel V., Le Bayon I., Gay-Perret P., Kutnik M., Fédérighi M. (2013). Methods for Recovering Microorganisms from Solid Surfaces Used in the Food Industry: A Review of the Literature. Int. J. Environ. Res. Public Health.

[87] Rawlinson S., Ciric L., Cloutman-Green E. (2019). How to carry out microbiological sampling of healthcare environment surfaces? A review of current evidence. J. Hosp. Infec..

[88] Plötze M., Niemz P. (2011). Porosity and pore size distribution of different wood types as determined by mercury intrusion porosimetry. Eur. J. Wood Prod..

[89] Cliver D.O. (2006). Cutting boards in Salmonella cross-contamination. J. AOAC Int..

[90] Schönwälder A., Kehr R., Wulf A., Smalla K. (2002). Wooden boards affecting the survival of bacteria?. Holz als Roh-und Werkstoff.

[91] Barnes B.I., Cassar C.A., Halablab M.A., Parkinson N.H., Miles R.J. (1996). An in situ method for determining bacterial survival on food preparation surfaces using a redox dye. Lett. Appl. Microb..

[92] Gupta M. Characterization of Microbial Contaminants Associated with Floor Material Types. https://www.semanticscholar.org/paper/Characterization-of-Microbial-Contaminants-with-Gupta/34640ec23e17fd96c6dab8a55afee6d5b154574e.

[93] Chai J., Donnelly T., Wong T., Bryce E. (2018). Environmental sampling of hospital surfaces: Assessing methodological quality. Canad. J. Infect. Control..

[94] Williams A.P., Avery L.M., Killham K., Jones D.L. (2005). Persistence of Escherichia coli O157 on farm surfaces under different environmental conditions. J. Appl. Microb..

[95] Coughenour C. (2009). An evaluation of methicillin resistant Staphylococcus aureus survival on five environmental surfaces under two different humidities, with and without the addition of bovine serum albumin. Master’s Thesis.

[96] Exum N.G., Kosek M.N., Davis M.F., Schwab K.J. (2017). Surface sampling collection and culture methods for Escherichia coli in household environments with high fecal contamination. Int. J. Environ. Res. Public Health.

[97] Lortal S., Di Blasi A., Madec M.-N., Pediliggieri C., Tuminello L., Tanguy G., Fauquant J., Lecuona Y., Campo P., Carpino S. (2009). Tina wooden vat biofilm: A safe and highly efficient lactic acid bacteria delivering system in PDO Ragusano cheese making. Int. J. Food Microb..

[98] Miller A.J., Brown T., Call J.E. (1996). Comparison of wooden and polyethylene cutting boards: Potential for the attachment and removal of bacteria from ground beef. J. Food Prot..

[99] Yoon H., Lee J.-Y., Suk H.-J., Lee S., Lee H., Lee S., Yoon Y. (2012). Modeling To Predict Growth/No Growth Boundaries and Kinetic Behavior of Salmonella on Cutting Board Surfaces. J. Food Prot..

[100] Copes J., Pellicer K., Malvestiti L., Stanchi N.O. Sobrevivencia en tablas de cocina de madera y plástico inoculadas experimentalmente con *Listeria monocytogenes. Survival of* Listeria monocytogenes *in cutting boards of plastic and wood experimentally contaminated*. http://sedici.unlp.edu.ar/handle/10915/11119.

[101] Boucher S.N., Chamberlain A.H.L., Adams M.R. (1998). Enhanced survival of Campylobacter jejuni in association with wood. J. Food Prot..

[102] Prechter S., Betz M., Cerny G., Wegener G., Windeisen E. (2002). Hygienische Aspekte von Schneidebrettern aus Holz bzw. Kunststoff. Holz als Roh-und Werkstoff.

[103] Baymiev A.K., Kuluev B.R., Shvets K.Y., Yamidanov R.S., Matniyazov R.T., Chemeris D.A., Zubov V.V., Alekseev Y.I., Mavzyutov A.R. (2020). Modern Approaches to Differentiation of Live and Dead Bacteria Using Selective Amplification of Nucleic Acids. Microbiology.

[104] Rozman U., Turk S.Š. (2016). PCR Technique for the Microbial Analysis of Inanimate Hospital Environment. Polym. Chain React. Biomed. Appl..

[105] Lee S., Bae S. (2018). Molecular viability testing of viable but non-culturable bacteria induced by antibiotic exposure. Microb. Biotech..

[106] Gibbs S.G., Sayles H., Colbert E.M., Hewlett A., Chaika O., Smith P.W. (2014). Evaluation of the Relationship between the Adenosine Triphosphate (ATP) Bioluminescence Assay and the Presence of Bacillus anthracis Spores and Vegetative Cells. Int. J. Environ. Res. Public Health.

[107] Nguyen D.T., Kim H.R., Jung J.H., Lee K.-B., Kim B.C. (2018). The development of paper discs immobilized with luciferase/D-luciferin for the detection of ATP from airborne bacteria. Sens. Actuat. B Chem..

[108] Raia D.D., Cannova L., Provenzano S., Santangelo O.E., Piazza D., Alagna E., Bonanno V., Aprea L., Firenze A. (2018). Comparison between adenosine triphosphate bioluminescence and aerobic colony count to assess surface sanitation in the hospital environment. Epidemiol. Biostat. Public Health.

[109] Bang J., Hong A., Kim H., Beuchat L.R., Rhee M.S., Kim Y., Ryu J.-H. (2014). Inactivation of Escherichia coli O157:H7 in biofilm on food-contact surfaces by sequential treatments of aqueous chlorine dioxide and drying. Int. J. Food Microb..

[110] Didienne R., Defargues C., Callon C., Meylheuc T., Hulin S., Montel M.-C. (2012). Characteristics of microbial biofilm on wooden vats (‘gerles’) in PDO Salers cheese. Int. J. Food Microb..

[111] Gaglio R., Cruciata M., Gerlando R.D., Scatassa M.L., Cardamone C., Mancuso I., Sardina M.T., Moschetti G., Portolano B., Settanni L. (2015). Microbial activation of wooden vats used for traditional cheese production and evolution of the neo-formed biofilms. Appl. Environ. Microbiol..

[112] Guillier L., Stahl V., Hezard B., Notz E., Briandet R. (2008). Modelling the competitive growth between Listeria monocytogenes and biofilm microflora of smear cheese wooden shelves. Int. J. Food Microb..

[113] Mariani C., Briandet R., Chamba J.-F., Notz E., Carnet-Pantiez A., Eyoug R.N., Oulahal N. (2007). Biofilm Ecology of Wooden Shelves Used in Ripening the French Raw Milk Smear Cheese Reblochon de Savoie. J. Dairy Sci..

[114] Scatassa M.L., Gaglio R., Macaluso G., Francesca N., Randazzo W., Cardamone C., Di Grigoli A., Moschetti G., Settanni L. (2015). Transfer, composition and technological characterization of the lactic acid bacterial populations of the wooden vats used to produce traditional stretched cheeses. Food Microb..

[115] Schubert M., Stührk C., Fuhr M.J., Schwarze F.W.M.R. (2014). Imaging hyphal growth of Physisporinus vitreus in Norway spruce wood by means of confocal laser scanning microscopy (CLSM). Holzforschung.

[116] Xiao Y., Wakeling R.N., Singh A.P. (2000). Use of confocal microscopy in examining fungi and bacteria in wood. Biofouling.

[117] Robson A.-L., Dastoor P.C., Flynn J., Palmer W., Martin A., Smith D.W., Woldu A., Hua S. (2018). Advantages and Limitations of Current Imaging Techniques for Characterizing Liposome Morphology. Front. Pharmacol..

[118] Fernández-Agulló A., Freire M.S., Ramírez-López C., Fernández-Moya J., González-Álvarez J. (2020). Valorization of residual walnut biomass from forest management and wood processing for the production of bioactive compounds. Biomass Conv. Bioref..

[119] Rowell R.M. (2012). Handbook of Wood Chemistry and Wood Composites.

[120] Hammud K.K., Neema R.R., Ali S.G., Hamza I.S. (2015). Direct Solid Disc as a Novel antibacterial testing method. Int. J. Adv. Pharm. Biol. Chem..

[121] Jain P., Shekhar N., Gaurav K. (2010). Antimicrobial activity and phytochemical analysis of Eucalyptus tereticornis bark and leaf methanolic extracts. Int. J. Pharm. Sci. Rev. Res..

[122] Nostro A., Germanò M.P., D’Angelo V., Marino A., Cannatelli M.A. (2000). Extraction methods and bioautography for evaluation of medicinal plant antimicrobial activity. Lett. Appl. Microb..

[123] Salem M.Z.M., Zayed M.Z., Ali H.M., El-Kareem M.S.M.A. (2016). Chemical composition, antioxidant and antibacterial activities of extracts from wood branch growing in Egypt. J. Wood Sci..

[124] Fentahun M., Yilkal B.A., Amsalu N., Alemayehu A., Amsalu G. (2017). Antibacterial Evaluation and Phytochemical Analysis of Selected Medicinal Plants against Some Pathogenic Enteric Bacteria in Gozamin District, Ethiopia. J. Pharmacovigil..

[125] Golus J., Sawicki R., Widelski J., Ginalska G. (2016). The agar microdilution method-a new method for antimicrobial susceptibility testing for essential oils and plant extracts. J. Appl. Microbiol..

[126] Dasgupta A., Krasowski M.D., Dasgupta A., Krasowski M.D. (2020). Chapter 10-Therapeutic drug monitoring of antimicrobial, antifungal and antiviral agents. Therapeutic Drug Monitoring Data (Fourth Edition).

[127] Dewanjee S., Gangopadhyay M., Bhattacharya N., Khanra R., Dua T.K. (2015). Bioautography and its scope in the field of natural product chemistry. J. Pharm. Anal..

[128] Choma I., Jesionek W. (2015). TLC-Direct Bioautography as a High Throughput Method for Detection of Antimicrobials in Plants. Chromatography.

[129] Masoko P., Masiphephethu M.V. (2019). Phytochemical Investigation, Antioxidant and Antimycobacterial Activities of Schkuhria pinnata (Lam) Thell Extracts Against Mycobacterium smegmatis. J. Evid.-Based Complement. Altern. Med..

[130] Suleimana M., McGaw L., Naidoo V., Eloff J. (2009). Detection of Antimicrobial Compounds by Bioautography of Different Extracts of Leaves of Selected South African Tree Species. Afr. J. Tradit. Complet. Altern. Med..

[131] Kovács J.K., Horváth G., Kerényi M., Kocsis B., Emődy L., Schneider G. (2016). A modified bioautographic method for antibacterial component screening against anaerobic and microaerophilic bacteria. J. Microb. Meth..

[132] Choma I.M., Grzelak E.M. (2011). Bioautography detection in thin-layer chromatography. J. Chromatol. A.

[133] Mai P.-Y., Levasseur M., Buisson D., Touboul D., Eparvier V. (2020). Identification of Antimicrobial Compounds from Sandwithia guyanensis-Associated Endophyte Using Molecular Network Approach. Plants.

[134] Mukai A., Takahashi K., Kofujita H., Ashitani T. (2019). Antitermite and antifungal activities of thujopsene natural autoxidation products. Eur. J. Wood Prod..

