# Supplementary material for: Testing the Antimicrobial Characteristics of Wood Materials: A Review of Methods
Source: Antibiotics (Basel). 2020 May 1;9(5):225. doi: 10.3390/antibiotics9050225 (PMC7277147; doi:10.3390/antibiotics9050225)
Supplement: Supplementary file 1 [file antibiotics-09-00225-s001.pdf]

antibiotics

MDPI

S1: Extraction methodologies to obtain the extractives from wood for antimicrobial activity testing

Wood species	Methodology	Reference
Sugi (*Cryptomeria japonica*)	Sawdust of heartwood subjected to Soxhlet extraction for 48 h using Methanol extract; fractionated with toluene and *n*-hexane to give solvent-soluble and solvent-insoluble fractions	[1]
T. arjuna	Dried bark powder was suspended in 50% or 90% ethanol for 1 or 7 days. After filtration and evaporation of ethanol, the extracts were oven dried at 60°C.	[2]
10 wood types	50–100g air dried powdered bark material was extracted with methanol at room temperature for 3 days and solvent was evaporated on a rotary-evaporator under reduced pressure at 55°C. The thick extracts dried in a hot-air oven at 50°C for 2 days.	[3]
Eucalyptus tereticornis	Twenty-five grams of the bark powder was soaked in 100 ml of methanol and allowed to stand for 24 hrs followed by boiling until the volume was reduced to one-third. The crude extracts were obtained by filtration and stored in a refrigerator at 4ºC.	[4]
13 trees not sure if the material was wood	40 g of sample was immersed in each of three conical flasks containing 400 ml of ethanol, methanol and chloroform solvent for 72 hours with shaking. After filtering via what man number 1 paper, the extract was further separated by rotary evaporator at 40°C reduced the temperature. Finally, the crude extract was placed in desiccators containing Cacl2.	[5]
P. rigida	Air dried heartwood was ground to 40–60 mesh and extracted in 80% Methanol @ 50g/150ml at room temperature in dark for 7 days. The solvents were evaporated using a rotary vacuum evaporator at 45 °C and dried extracts were stored at 4 °C.	[6]
Schinus molle	100 g dried powder of woody branch added in 250 mL of each solvent, Essential oils, methanol (ME), dichloromethane (DCME) and water (WE) extracts and the solvents were evaporated to dryness using a rotary evaporator.	[7]
Pinus sylvestris and *Picea abies L.*	Untreated and thermally modified oven dried wood samples were extracted with acetone using a Soxhlet apparatus for 6 h. The acetone-soluble extractive content was measured from approximately 10 g of drilled wood at ambient temperature. Acetone was evaporated from the samples using a rotary evaporator. The extractive content was calculated based on dry wood weight.	[8–10]
Eucalyptus (*Eucalyptus globulus*) Walnut (*Juglans regia L.)*	Wood veneer trimmings were extracted by two methods, maceration in an orbital bath and microwave-assisted extraction. In the conventional one, the effect of solvent (water, MeOH, EtOH, 50% MeOH and 50% EtOH), temperature (50 and 75 °C) and particle size on extraction yield and extract properties were analyzed. The microwave method was used to study the influence of temperature (50-70 °C), the liquid-solid ratio (5:1-10:1 mL/g) and time (5-15 min) on extraction yield and extract properties.	[11,12]
30 species of hard and soft wood trees	Accelerated solvent extractor (ASE) apparatus was used and the lipophilic extractives were first extracted with hexane, and thereafter the hydrophilic extractives were extracted with an acetone/water (95:5 v/v) mixture.	[13]
Oaks, chestnut, vine, and cherry	Pressurized liquid extraction of oenological wood was carried out by means of an accelerated solvent extractor. Five grams of sawdust, dispersed in 2 g of diatomaceous earth, was placed into inox extraction cells of 11 mL, which was filled with a mixture of methanol/water (50:50) as extracting solvent.	[14]

Mango wood *Mangifera indica (L.)*	500 g air-dried bark powder was first extracted with 600 mL n-hexane in a Soxhlet for 48 h. The solvent was removed and the residue (15 g) was kept aside. The extracted powder was dried in air and re-extracted with methanol in soxhlet for 30 h. Again, the solvent was removed and the residue (13 g) was collected. Both extracts were stored at 4 °C in a refrigerator for further use.	[15]
Pine, cicas, and thoja	Stem powder packed in watman paper 1 extracted by Soxhlet apparatus using petroleum ether, ethanol, chloroform and water.	[16]
Kiam wood (*C. lanceotatum*)	The pieces of Kiam wood were mixed with water in a proportion of 1g: 100 mL. The extraction was performed by continuous stirring in a water bath at 50 C for 24 h. The extract thus obtained was filtered through Whatman no. 1 filter paper and dried by using a hot air oven at 50 C for 24 h, ground and placed in a bottle at 4 C until needed.	[17]
Xylia xylocarpa (Roxb.) Taub.	sawdust was extracted with chloroform-methanol 1:1 ratio (v/v) and separated to 4 fractions with hexane, dichloromethane, ethyl acetate and 30% methanol	[18].
Multiple wood	We obtained the essential oil, hydrosol, distillation residue, and distillation wastewater from the trees.	[19]
Larch and Pine	MeOH-extracts of sawdust and wood plates @ (1 g of wood to 10 mL of MeOH for 24 h at room temperature) were produced for each kind of wood	[20]
Walnut (*J. regia*)	10 gram bark and mixing it in 100 mL water overnight. Next day, the suspension was incubated in boiling water bath for two minutes. Subsequently, the mixture was cooled, centrifuged at 2500 g, and the supernatant was filtered through 0.2 µm filter. The filtrate was evaporated using a refrigerated CentriVap Concentrator evaporator until dry and stored at 4°C until further use.	[21]
Beech wood (*Fagus sylvatica* L.)	Wound-associated extracts were spectrophotometrically analyzed and a paper disc screening test was applied to estimate their fungicidal potential against selected brown (*Gloeophyllum trabeum*) and white (*Trametes versicolor*) rot fungi.	[22]
Poplar wood	The samples were extracted by aqueous extract and 70% ethanol solution twice by the hot reflux method. The first duration of extraction was 5 h and the solid-liquid ratio was 1:30. The duration of the second extraction was 3 h and the solid-liquid ratio was 1:15. The wood extracts of the first and second extractions were filtrated and pooled, and the solvent was recovered in a water bath using a rotary evaporator to calculate the extracts yield.	[23]
Picea abies, Larix decidua	The extracts were prepared from ground air-dried (40 to 60 mesh) wood and bark samples, and extracted three times using 95% methanol over a water bath for one day at room temperature.	[24]

S2: Microbial recovery protocols applied on wood materials in different studies

Wood	Bacteria	Methods	Main findings of study	Study
**Hardwood floor**	*Staphylococcus aureus*, *Aspergillus niger*	Contact, Vacuum, and Bulk rinsate	Microbial survival depends on recovery method and surface type in hospitals (vet and human) and office buildings	[28]
**Pine**<br>**Polar**<br>**Spruce**	*Escherichia coli*,<br>*Listeria monocytogenes*,<br>*Penicillium expansum*	Planning<br>Grinding<br>Brushing	Humidity, type of wood, type of microbe and recovery method influence the recovery rate of microbes	[29]
**Archaeological objects**	*L. monocytogenes, Pe. Chrysogenum, A. niger, Corynebacterium pyogenes*	Swabbing	Qualitative results of microbes responsible for wood degradation were identified	[25]
**Larch Shavings**	*Klebsiella pneumoniae*, MRSA	Blotting and Vibration	Microbial quantities decreased after contact with wood	[26]
**Poplar**	*E. coli* and *P. expansum*	Grinding/blending	There is low transmission of microbes from wood to food	[27]
	E. coli	Dry cloth method	A cheaper method used on 6-floor materials	[30]
**Spruce**	*L. monocytogenes*	Surface contact /blot<br>Planning and Blending	L mono cannot be cleaned by brushing and rubbing	[31]
**painted wood panels**	*Bacillus anthracis*	Sponge	Method tested on 6 surfaces	[32]
**Hard maple cutting board**	*E. coli*	Wet sponge Swabbing	Microbial recovery was 0.25 and 0.1 % from plastic and wood respectively in dry conditions and the difference was non significant in wet conditions	[33]
**Maple and Beech**	Aerobic mesophilic microorganisms and *Enterobacteriaceae*, and *Pseudomonas* spp.	Swabbing	Survival of microbes on different cutting boards before and after cleaning	[34]
**Cork oak**	*E. coli, S. aureus*	Shaking/stirring to recover microbes	AATCC-100-2004 method of textile products	[35]
**Cutting boards**	*V. parahaemolyticus*	Stirring the inoculated piece of wood in alkaline peptone water	More number of microbes were recovered from plastic as compared to wood and bamboo samples	[36]
**Wooden toothpicks**	*S. aureus, E. coli, Pseudomonas aeruginosa, Proteus and Salmonella,*	Sample in solution and Centrifuged to isolate bacterial cells	Microbial contamination were found in multiple samples	[37]

	Giardia lambia and Ascaris lumbricoides			
**Wooden cutting boards**	*E. coli*	Swabbing	Efficacy of Cleaning methods on meat cutting boards	[38]
**Maple and oak of different ages**	*E. coli*	Shaking the inoculated material by shaker and vortex	Persistence of bacteria on different surfaces in farm conditions	[39]
**Mango, teak, Tamarind**	*Salmonella strains, E.coli*	Swabbing	Qualitative analysis showed presence of microbes on utensils after 24 hours	[40]
**Wooden cutting board**	*E. coli* and *S. aureus*	Wiping	Microbial survival time was least on wood	[41]
**Wood**	*Salmonella* Typhimurium	Swabbing (vortexting), Contact pressing (635 g) and food contact	Number of microbes recovered and their transfer from wood to food was lowest as compared to other surfaces	[42]
**Wood and other cutting boards**	*S.* enteritidis	Swabbing and Contact press	Efficacy of cleaning methods was tested	[43]
**5 wood crates and Oak**	*LABS*	Swabbing	Wine making	[44]
**Wood cutting board and other surfaces**	*MRSA*	Swabbing	AMR on hospital surfaces	[45,46]
**Wood applicator sticks**	*Shigella sonnei*	Gentle shaking of inoculated wood piece in PBS	Bacteria survived on wood at different temperatures for a long time period	[47]
**Chestnut, douglas**	*Lactic Acid Bacteria*	Brushing	LAB were dominant organism forming biofilms on cheese making boards	[48]
**Maple, beech**	*Aerobic mesophilic & Enterobacteriaceae*	Swabbing	No problem of cleaning the wood surfaces	[34]
**Rubber wood**	*L.monocytogenes*	Rinsing with normal saline	Transmission of Listeria monocytogenes from raw chicken meat to cooked chicken meat through cutting boards	[49]
**Rubber wood**	*Campylobacter jejuni*	Rinsing with normal saline and then counting CFU by combined most-probable-number (MPN)-PCR method	Transfer during uncooked/cooked meat chopping on unscored and scored cutting boards	[50]

**Hardwood**	*Salmonella* Typhurieum	Food contact cross contamination and then stomaching the contaminated food to recover bacteria	Contact time and contamination	[51]
**Scots pine, Norway spruce, European larch, beech, Black poplar**	*E. coli* pIE639 and *Enterococcus faecium*	Direct contact	Wood pressed on agar for 10-20 seconds	[52]
**Pine**	*E. coli, P. aeruginosa, S. aureus, L. monocytogenes*	Swabbing	Treatment was efficient for reducing microbial contamination on plastic and wooden cutting boards	[53]
**Poplar**	*Salmonella*	Swabbing	Modelling of salmonella growth behavior on wood boards	[54]
**Beech**	*Campylobacter*	Swabbing	Killing of bacteria by glycerol monocaprate	[55]
**Birch**	*L. monocytogenes*	Manual shaking	Survival of bacteria on the wood surfaces in contact with salmon fish	[56]
**Cutting board**	*L. monocytogenes*	Swabbing, and blotting	Listeria survived for more than 2 months on different cutting board surfaces	[57]
**Maple wood And other surfaces**	*Enterobacter aerogenes*	Vortex	CFU counting	[58]
**Wooden boards**	*Multiple* bacteria	Swabbing	Cheese making process	[59]
**Wooden boards**	*Multiple* bacteria	Brushing	Cheese making	[60]
**Spruce fir boards (*Picea abies*)**	*Listeria monocytogenes, Listeria innocua*	Planning (vertical drill press @ 100 rpm) and cotton swabbing and then stomacher blender	Heating ensured *Listeria* free hygienic status of the wood. A comparison of abrasive (shavings) and swabbing (cotton rolls) sampling methods resulted in identical results.	[61]
**Wood laminate**	*Erwinia herbicola*	Sponge and a macrofoam swab	Culture method showed because very low numbers of cells (0.7 to 52.2%) were isolated so quantitative PCR (QPCR) amplification assay was used	[62]
**Poplar**	*Bacillus cereus* spores and *E. coli* cells	Direct contact (wood in broth)	Impedance analysis of microbes in contact with wood present in broth showed decrease in microbial count	[63]
**Poplar and pine**	Total microbial counts	Vortexing the contaminated pieces	Microbes decreased fastest on wood	[64]

References

1. 1. Matsushita, Y.; Hwang, Y.-H.; Sugamoto, K.; Matsui, T. Antimicrobial activity of heartwood components of sugi (<Emphasis Type="Italic">Cryptomeria japonica</Emphasis>) against several fungi and bacteria. *J. Wood Sci.* **2006**, *52*, 552–556.
2. 2. Khan, R.; Islam, B.; Akram, M.; Shakil, S.; Ahmad, A.A.; Ali, S.M.; Siddiqui, M.; Khan, A.U. Antimicrobial Activity of Five Herbal Extracts Against Multi Drug Resistant (MDR) Strains of Bacteria and Fungus of Clinical Origin. *Molecules* **2009**, *14*, 586–597.
3. 3. Khan, M.N.; Ngassapa, O.; Matee, M.I.N. Antimicrobial Activity of Tanzanian Chewing Sticks Against Oral Pathogenic Microbes. *Pharm. Biol.* **2000**, *38*, 235–240.
4. 4. Jain, P.; Shekhar, N.; Gaurav, K. Antimicrobial activity and phytochemical analysis of Eucalyptus tereticornis bark and leaf methanolic extracts. *Int. J. Pharm. Sci. Rev. Res.* **2010**, *4*, 126–128.
5. 5. Fentahun, M.; Yilkal B, A.; Amsalu, N.; Alemayehu, A.; Amsalu, G. Antibacterial Evaluation and Phytochemical Analysis of Selected Medicinal Plants against Some Pathogenic Enteric Bacteria in Gozamin District, Ethiopia. *J. Pharmacovigil.* **2017**, *5*, 244.
6. 6. Salem, M.Z.M.; Zidan, Y.E.; El Hadidi, N.M.N.; Mansour, M.M.A.; Abo Elgat, W.A.A. Evaluation of usage three natural extracts applied to three commercial wood species against five common molds. *Int. Biodeterior. Biodegrad.* **2016**, *110*, 206–226.
7. 7. Salem, M.Z.M.; Zayed, M.Z.; Ali, H.M.; El-Kareem, M.S.M.A. Chemical composition, antioxidant and antibacterial activities of extracts from <Emphasis Type="Italic">Schinus molle</Emphasis> wood branch growing in Egypt. *J. Wood Sci.* **2016**, *62*, 548–561.
8. 8. Vainio-Kaila, T.; Rautkari, L.; Nordström, K.; Närhi, M.; Natri, O.; Kairi, M. Effect of extractives and thermal modification on antibacterial properties of Scots pine and Norway spruce. *Int. Wood Prod. J.* **2013**, *4*, 248–252.
9. 9. Vainio-Kaila, T.; Kyyhkynen, A.; Rautkari, L.; Siitonen, A. Antibacterial Effects of Extracts of Pinus sylvestris and Picea abies against Staphylococcus aureus, Enterococcus faecalis, Escherichia coli, and Streptococcus pneumoniae. *BioResources* **2015**, *10*, 7763–7771.
10. 10. Vainio-Kaila, T.; Zhang, X.; Hänninen, T.; Kyyhkynen, A.; Johansson, L.-S.; Willför, S.; Österberg, M.; Siitonen, A.; Rautkari, L. Antibacterial Effects of Wood Structural Components and Extractives from Pinus sylvestris and Picea abies on Methicillin-Resistant Staphylococcus aureus and Escherichia coli O157:H7. *BioResources* **2017**, *12*, 7601–7614.
11. 11. Fernández-Agulló, A.; Pereira, E.; Freire, M.S.; Valentão, P.; Andrade, P.B.; González-Álvarez, J.; Pereira, J.A. Influence of solvent on the antioxidant and antimicrobial properties of walnut (Juglans regia L.) green husk extracts. *Ind. Crops Prod.* **2013**, *42*, 126–132.
12. 12. Fernández-Agulló, A.; Freire, M.S.; González-Álvarez, J. Effect of the extraction technique on the recovery of bioactive compounds from eucalyptus (Eucalyptus globulus) wood industrial wastes. *Ind. Crops Prod.* **2015**, *64*, 105–113.
13. 13. Valimaa, A.-L.; Honkalampi-Hämäläinen, U.; Pietarinen, S.; Willför, S.; Holmbom, B.; von Wright, A. Antimicrobial and cytotoxic knotwood extracts and related pure compounds and their effects on food-associated microorganisms. *Int. J. Food Microbiol.* **2007**, *115*, 235–243.
14. 14. Alañón, M.E.; García-Ruíz, A.; Díaz-Maroto, M.C.; Pérez-Coello, M.S.; Moreno-Arribas, M.V. Antimicrobial and antioxidant activity of pressurized liquid extracts from oenological woods. *Food Control* **2015**, *50*, 581–588.
15. 15. Singh, R.; Singh, S.K.; Maharia, R.S.; Garg, A.N. Identification of new phytoconstituents and antimicrobial activity in stem bark of Mangifera indica (L.). *J. Pharm. Biomed. Anal.* **2015**, *105*, 150–155.
16. 16. Bissa, S.; Bohra, A.; Bohra, A. Antibacterial potential of three naked-seeded (Gymnosperm) plants. **2008**.
17. 17. Chana-Thaworn, J.; Chanthachum, S.; Wittaya, T. Properties and antimicrobial activity of edible films incorporated with kiam wood (Cotyleobium lanceotatum) extract. *LWT - Food Sci. Technol.* **2011**, *44*, 284–292.

18. 18. Nakmee, P.S.; Khuntong, S.; Nuengchamnong, N. Phytochemical Constituents with Antimicrobial and Antioxidant Activities from Xylia xylocarpa (Roxb.) Taub. Sawdust Extracts. *Chiang Mai J. Sci.* **2016**, *43*, 11–21.
19. 19. Nakagawa, T.; Zhu, Q.; Ishikawa, H.; Ohnuki, K.; Kakino, K.; Horiuchi, N.; Shinotsuka, H.; Naito, T.; Matsumoto, T.; Minamisawa, N.; et al. Multiple uses of Essential Oil and By-Products from Various Parts of the Yakushima Native Cedar (Cryptomeria Japonica). *J. Wood Chem. Technol.* **2016**, *36*, 42–55.
20. 20. Laireiter, C.M.; Schnabel, T.; Köck, A.; Stalzer, P.; Petutschnigg, A.; Oostingh, G.J.; Hell, M. Active Anti-Microbial Effects of Larch and Pine Wood on Four Bacterial Strains. *BioResources* **2013**, *9*, 273–281.
21. 21. Iqbal, J.; Siddiqui, R.; Kazmi, S.U.; Khan, N.A. A Simple Assay to Screen Antimicrobial Compounds Potentiating the Activity of Current Antibiotics Available online: https://www.hindawi.com/journals/bmri/2013/927323/ (accessed on Mar 22, 2018).
22. 22. Vek, V.; Oven, P.; Humar, M. Phenolic extractives of wound-associated wood of beech and their fungicidal effect. *Int. Biodeterior. Biodegrad.* **2013**, *77*, 91–97.
23. 23. Cai, M.; Lv, H.; Cao, C.; Zhang, L.; Cao, R.; Xu, B. Evaluation of antimicrobial activity of Pterocarpus extracts. *Ind. Crops Prod.* **2019**, *140*, 111668.
24. 24. Salem, M.Z.M.; Elansary, H.O.; Elkelish, A.A.; Zeidler, A.; Ali, H.M.; EL-Hefny, M.; Yessoufou, K. In vitro Bioactivity and Antimicrobial Activity of Picea abies and Larix decidua Wood and Bark Extracts. *BioResources* **2016**, *11*, 9421–9437.
25. 25. Elserogy, A.; Kanan, G.; Hussein, E.; Khreis, S.A. Isolation, characterization and treatment of microbial agents responsible for the deterioration of archaeological objects in three jordanian museums. *Mediterr. Archa Eology Archaeom.* **2016**, *16*, 117–126.
26. 26. Kavian-Jahromi, N.; Schagerl, L.; Dürschmied, B.; Enzinger, S.; Schnabl, C.; Schnabel, T.; Petutschnigg, A. Comparison of the antibacterial effects of sapwood and heartwood of the larch tree focusing on the use in hygiene sensitive areas. *Eur. J. Wood Wood Prod.* **2015**, *73*, 841–844.
27. 27. Montibus, M.; Ismaïl, R.; Michel, V.; Federighi, M.; Aviat, F.; Le Bayon, I. Assessment of Penicillium expansum and Escherichia coli transfer from poplar crates to apples. *Food Control* **2016**, *60*, 95–102.
28. 28. Gupta, M. Characterization of Microbial Contaminants Associated with Floor Material Types, The Ohio State University, 2017.
29. 29. Ismail, R.; Bayon, I.L.; Michel, V.; Jequel, M.; Kutnik, M.; Aviat, F.; Fédérighi, M. Comparative Study of Three Methods for Recovering Microorganisms from Wooden Surfaces in the Food Industry. *Food Anal. Methods* **2015**, *8*, 1238–1247.
30. 30. Exum, N.G.; Kosek, M.N.; Davis, M.F.; Schwab, K.J. Surface sampling collection and culture methods for Escherichia coli in household environments with high fecal contamination. *Int. J. Environ. Res. Public. Health* **2017**, *14*.
31. 31. Zangerl, P.; Matlschweiger, C.; Dillinger, K.; Eliskases-Lechner, F. Survival of Listeria monocytogenes after cleaning and sanitation of wooden shelves used for cheese ripening. *Eur. J. Wood Wood Prod.* **2010**, *68*, 415–419.
32. 32. Piepel, G.F.; Amidan, B.G.; Krauter, P.; Einfeld, W. *Experimental Design for a Sponge-Wipe Study to Relate the Recovery Efficiency and False Negative Rate to the Concentration of a Bacillus anthracis Surrogate for Six Surface Materials*; Pacific Northwest National Lab. (PNNL), Richland, WA (United States), 2010;
33. 33. Welker, C.; Faiola, N.; Davis, S.; Maffatore, I.; Batt, C.A. Bacterial Retention and Cleanability of Plastic and Wood Cutting Boards with Commercial Food Service Maintenance Practices. *J. Food Prot.* **1997**, *60*, 407–413.
34. 34. Lucke, F.-K.; Skowyrska, A. Hygienic aspects of using wooden and plastic cutting boards, assessed in laboratory and small gastronomy units. *J. Für Verbraucherschutz Leb.* **2015**, *10*, 317–322.
35. 35. Gonçalves, F.; Correia, P.; Silva, S.P.; Almeida-Aguiar, C. Evaluation of antimicrobial properties of cork. *FEMS Microbiol. Lett.* **2016**, *363*.
36. 36. Chiu, T.-H.; Duan, J.; Liu, C.; Su, Y.-C. Efficacy of electrolysed oxidizing water in inactivating Vibrio parahaemolyticus on kitchen cutting boards and food contact surfaces. *Lett. Appl. Microbiol.* **2006**, *43*, 666–672.
37. 37. Elom, M.O.; Ugah, U.I.; Omote, V. Microbial Contaminants of Wooden Toothpicks in Abakaliki Metropolis, Ebonyi State, Nigeria. *World J. Life Sci. Med. Res.* **2014**, *3*, 101.
38. 38. Miller, A.J.; Brown, T.; Call, J.E. Comparison of wooden and polyethylene cutting boards: Potential for the attachment and removal of bacteria from ground beef. *J. Food Prot.* **1996**, *59*, 854–858.

39. 39. Williams, A.P.; Avery, L.M.; Killham, K.; Jones, D.L. Persistence of Escherichia coli O157 on farm surfaces under different environmental conditions. *J. Appl. Microbiol.* **2005**, *98*, 1075–1083.
40. 40. Suresh, T.; Srinivasan, D.; Hatha, A.A.M.; Lakshmanaperumalsamy, P. The Incidence, Antibiotic Resistance and Survival of Salmonella and Escherichia coli Isolated from Broiler Chicken Retail Outlets. *Microbes Environ.* **2000**, *15*, 173–181.
41. 41. DeVere, E.; Purchase, D. Effectiveness of domestic antibacterial products in decontaminating food contact surfaces. *Food Microbiol.* **2007**, *24*, 425–430.
42. 42. Moore, G.; Blair, I.S.; McDowell, D.A. Recovery and transfer of Salmonella Typhimurium from four different domestic food contact surfaces. *J. Food Prot.* **2007**, *70*, 2273–2280.
43. 43. Soares, V.M.; Pereira, J.G.; Viana, C.; Izidoro, T.B.; Bersot, L. dos S.; Pinto, J.P. de A.N. Transfer of Salmonella Enteritidis to four types of surfaces after cleaning procedures and cross-contamination to tomatoes. *Food Microbiol.* **2012**, *30*, 453–456.
44. 44. Swaffield, C.H.; Scott, J.A.; Jarvis, B. Observations on the microbial ecology of traditional alcoholic cider storage vats. *Food Microbiol.* **1997**, *14*, 353–361.
45. 45. Coughenour, C. An evaluation of methicillin resistant Staphylococcus aureus survival on five environmental surfaces under two different humidities, with and without the addition of bovine serum albumin. *UNLV Theses Diss. Prof. Pap. Capstones* **2009**.
46. 46. Coughenour, C.; Stevens, V.; Stetzenbach, L.D. An Evaluation of Methicillin-Resistant Staphylococcus aureus Survival on Five Environmental Surfaces. *Microb. Drug Resist.* **2011**, *17*, 457–461.
47. 47. Nakamura, M. The survival of Shigella sonnei on cotton, glass, wood, paper, and metal at various temperatures*. *Epidemiol. Amp Infect.* **1962**, *60*, 35–39.
48. 48. Scatassa, M.L.; Gaglio, R.; Macaluso, G.; Francesca, N.; Randazzo, W.; Cardamone, C.; Di Grigoli, A.; Moschetti, G.; Settanni, L. Transfer, composition and technological characterization of the lactic acid bacterial populations of the wooden vats used to produce traditional stretched cheeses. *Food Microbiol.* **2015**, *52*, 31–41.
49. 49. Goh, S.G.; Leili, A.-H.; Kuan, C.H.; Loo, Y.Y.; Lye, Y.L.; Chang, W.S.; Soopna, P.; Najwa, Mohd.S.; Tang, J.Y.H.; Yaya, R.; et al. Transmission of Listeria monocytogenes from raw chicken meat to cooked chicken meat through cutting boards. *Food Control* **2014**, *37*, 51–55.
50. 50. Tang, J.Y.H.; Nishibuchi, M.; Nakaguchi, Y.; Ghazali, F.M.; Saleha, A.A.; Son, R. Transfer of Campylobacter jejuni from raw to cooked chicken via wood and plastic cutting boards. *Lett. Appl. Microbiol.* **2011**, *52*, 581–588.
51. 51. Dawson, P.; Han, I.; Cox, M.; Black, C.; Simmons, L. Residence time and food contact time effects on transfer of Salmonella Typhimurium from tile, wood and carpet: testing the five-second rule. *J. Appl. Microbiol.* **2007**, *102*, 945–953.
52. 52. Schönwälder, A.; Kehr, R.; Wulf, A.; Smalla, K. Wooden boards affecting the survival of bacteria? *Holz Als Roh- Werkst.* **2002**, *60*, 249–257.
53. 53. Deza, M.A.; Araujo, M.; Garrido, M.J. Efficacy of Neutral Electrolyzed Water To Inactivate Escherichia coli, Listeria monocytogenes, Pseudomonas aeruginosa, and Staphylococcus aureus on Plastic and Wooden Kitchen Cutting Boards. *J. Food Prot.* **2007**, *70*, 102–108.
54. 54. Yoon, H.; Lee, J.-Y.; Suk, H.-J.; Lee, S.; Lee, H.; Lee, S.; Yoon, Y. Modeling To Predict Growth/No Growth Boundaries and Kinetic Behavior of Salmonella on Cutting Board Surfaces. *J. Food Prot.* **2012**, *75*, 2116–2121.
55. 55. Thormar, H.; Hilmarsson, H. Killing of Campylobacter on contaminated plastic and wooden cutting boards by glycerol monocaprate (monocaprin). *Lett. Appl. Microbiol.* **2010**, *51*, 319–324.
56. 56. Hsu, J.-L.; Opitz, H.M.; Bayer, R.C.; Kling, L.J.; Halteman, W.A.; Martin, R.E.; Slabyj, B.M. Listeria monocytogenes in an Atlantic Salmon (Salmo salar) Processing Environment. *J. Food Prot.* **2005**, *68*, 1635–1640.
57. 57. Copes, J.; Pellicer, K.; Malvestiti, L.; Stanchi, N.O. Sobrevivencia en tablas de cocina de madera y plástico inoculadas experimentalmente con *Listeria monocytogenes*. *Analecta Vet.* **2000**, *20, no. 2*.
58. 58. Miranda, R.C.; Schaffner, D.W. Longer Contact Times Increase Cross-Contamination of Enterobacter aerogenes from Surfaces to Food. *Appl. Environ. Microbiol.* **2016**, *82*, 6490–6496.
59. 59. Lortal, S.; Di Blasi, A.; Madec, M.-N.; Pediliggieri, C.; Tuminello, L.; Tanguy, G.; Fauquant, J.; Lecuona, Y.; Campo, P.; Carpino, S.; et al. Tina wooden vat biofilm: A safe and highly efficient lactic acid bacteria delivering system in PDO Ragusano cheese making. *Int. J. Food Microbiol.* **2009**, *132*, 1–8.

60. 60. Didienne, R.; Defargues, C.; Callon, C.; Meylheuc, T.; Hulin, S.; Montel, M.-C. Characteristics of microbial biofilm on wooden vats ('gerles') in PDO Salers cheese. *Int. J. Food Microbiol.* **2012**, *156*, 91–101.
61. 61. Imhof, R.; Schwendimann, L.; Scettrini, P.R. Sanitising wooden boards used for cheese maturation by means of a steam-mediated heating process. *J. Consum. Prot. Food Saf.* **2017**, *12*, 255–263.
62. 62. Buttner, M.P.; Cruz, P.; Stetzenbach, L.D.; Cronin, T. Evaluation of Two Surface Sampling Methods for Detection of Erwinia herbicola on a Variety of Materials by Culture and Quantitative PCR. *Appl. Environ. Microbiol.* **2007**, *73*, 3505–3510.
63. 63. Revol-Junelles, A.-M.; Miguindou-Mabiala, R.; Roger-Maigné, D.; Millière, J.-B. Behavior of Escherichia coli cells and Bacillus cereus spores on poplar wood crates by impedance measurements. *J. Food Prot.* **2005**, *68*, 80–84.
64. 64. Ripolles-Avila, C.; Hascoët, A.S.; Ríos-Castillo, A.G.; Rodríguez-Jerez, J.J. Hygienic properties exhibited by single-use wood and plastic packaging on the microbial stability for fish. *LWT* **2019**, *113*, 108309.

© 2020 by the authors. Submitted for possible open access publication under the terms and conditions of the Creative Commons Attribution (CC BY) license (http://creativecommons.org/licenses/by/4.0/).
